# The transmembrane supercomplex mediating the biogenesis of OMPs in Gram‐negative bacteria assumes a circular conformational change upon activation

**DOI:** 10.1002/2211-5463.12922

**Published:** 2020-07-23

**Authors:** Feng Jin

**Affiliations:** ^1^ School of Life Sciences Peking University Beijing China; ^2^ Peking‐Tsinghua Center for Life Sciences Peking University Beijing China

**Keywords:** BAM complex, biogenesis of OMPs, protein modeling, supercomplex, SurA

## Abstract

The cell envelope of Gram‐negative bacteria is composed of the inner (plasma) and the outer membrane. In the outer membrane, the outer membrane β‐barrel proteins (OMPs) serve multiple functions. They are synthesized in the cytoplasm and finally inserted into the outer membrane through a critical and complex pathway facilitated by many protein factors. Recently, a new model for the biogenesis of OMPs in Gram‐negative bacteria was proposed, in which a supercomplex containing multiple proteins spans the inner and outer membrane, to mediate the biogenesis of OMPs. The core part of the transmembrane supercomplex is the inner membrane protein translocon and the outer membrane β‐barrel assembly machinery (BAM) complex. Some components of the supercomplex, such as the BamA subunit of the BAM complex, are essential and conserved across species. The other components, for example, the BamB subunit and the primary periplasmic chaperone SurA, are also required for the supercomplex to gain complete function and full efficiency. How BamB and SurA behave in the supercomplex, however, is less well understood. Therefore, the crosstalk between BamA, BamB and SurA was investigated mainly through *in vivo* protein photo‐cross‐linking experiments and protein modeling. Moreover, theoretical structures for part of the supercomplex consisting of SurA and the BAM complex were constructed. The modeling data are consistent with the experimental results. The theoretical structures computed in this work provide a more comprehensive view of the mechanism of the supercomplex, demonstrating a circular conformational change of the supercomplex when it is active.

## Graphical abstract

I present here the interaction network among the subunits of the β‐barrel assembly machinery (BAM) complex (BamA, BamB) and the periplasmic chaperone SurA, as well as the existence of a ternary BamA–BamB–SurA complex. Theoretical structures for the SurA‐BAM complex, which is part of the transmembrane supercomplex involved in the biogenesis of OMPs in Gram‐negative bacteria, were computed to interpret the mechanism.

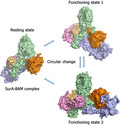

**Abbreviations**
BAM: β‐barrel assembly machinery
OMP: outer membrane β‐barrel protein
pBpa: *p*‐benzoyl‐l‐phenylalanine
PDB: Protein Data Bank
POTRA: polypeptide transport‐associated

Gram‐negative bacteria, as well as mitochondria and chloroplasts, which are eukaryotic organelles, contain two membranes, the inner (plasma) and the outer membrane [1]. The outer membrane β‐barrel proteins (OMPs), adopting a unique barrel‐like topology composed of β‐sheets [2], serve multiple functions in the outer membrane [1, 3]. In Gram‐negative bacteria, the OMPs are targeted to the outer membrane through a vital and long pathway related to multiple protein factors [4, 5, 6]. The OMPs are synthesized in the cytoplasm and are proposed to be translocated across the inner membrane to the periplasm (the space between the inner and outer membrane) through the *sec* translocon [4, 6] via a ‘lateral gate’ model [7, 8]. In prokaryotic cells, the core part of the *sec* translocon is the heterotrimeric SecYEG protein complex embedded in the inner membrane [9, 10]. In the currently prevailing model, the SecYEG complex is believed to form the protein‐conducting channel in the inner membrane, and the SecA protein pushes the nascent OMPs passing through the channel [7, 8]. SecY/E and SecA are essential and highly conserved [10, 11]. SecA is an ATPase found in the cytoplasm [12]. However, a small portion of SecA has also been demonstrated to be inserted in the membrane [13, 14] or even exposed to the periplasm [15]. SecA could translocate OMPs without SecY/E [16, 17]. Moreover, SecA and SecA fragments are able to form a porelike structure in the membrane, implying that they may form the core of bacterial protein‐conducting channels [18]. The following folding and membrane integration steps require the participation of periplasmic chaperones [19, 20] and the β‐barrel assembly machinery (BAM; β‐barrel protein assembly machine) complex [4, 5, 21, 22, 23, 24]. It is commonly believed that after translocation, the nascent OMPs are escorted by the periplasmic chaperones to the BAM complex, and then the OMPs are folded and integrated into the membrane by the BAM complex [4, 5]. Recently, it was revealed that a supercomplex containing multiple protein factors, including SecA, SecY, periplasmic chaperone SurA, subunits of BAM complex and so forth, spans the inner and outer membrane to mediate the biogenesis of OMPs [25]. The supercomplex could integrate the translocation, transport, folding and membrane insertion of the OMPs, but the mechanism is still less understood.

The core part of the supercomplex is composed of the inner membrane translocon and the outer membrane BAM complex [25, 26]. The BAM complex plays an essential role in the folding and membrane integration of OMPs [21, 22, 23, 24]. The core subunit of the BAM complex, BamA (also named as YaeT/Omp85), is an OMP belonging to the Omp85 protein superfamily [21]. BamA in Gram‐negative bacteria and its homologs, Sam50 in mitochondria and Toc75 in chloroplast, are essential for the cell growth; moreover, they have been demonstrated to directly serve an essential function in the biogenesis of OMPs [21, 22, 23, 24, 27]. BamA is composed of the N‐terminal polypeptide transport‐associated (POTRA) domains [28] and the C‐terminal transmembrane domain [21, 22, 23, 24]. The C‐terminal transmembrane domain is highly conserved and essential, but the number and the essentiality of POTRA domains vary across species [29, 30, 31]. Commonly in Gram‐negative bacteria, BamA contains five POTRA domains located in the periplasm [28, 29, 30, 31] with low sequence identity but similar structure [30], and might be involved in the transport of client proteins or act as the scaffold for the other subunits of the BAM complex [24, 30, 31]. The β‐barrel domain of BamA is proposed to function as the channel for client proteins and may open laterally to secret client proteins into the outer membrane [24, 32, 33, 34].

Besides BamA, other subunits of the BAM complex are also required to gain complete function and full efficiency in the biogenesis of OMPs, although they are not as highly conserved as BamA [27]. In *Escherichia coli*, the BAM complex is composed of BamA and four outer membrane lipoproteins (BamB/YfgL, BamC/NlpB, BamD/YfiO and BamE/SmpA) attached to the outer membrane through the anchor at the N terminus. Most BAM complexes in Gram‐negative bacteria contain BamB‐E as that in *E. coli*, but certain subunits are missing or substituted by equivalents in certain species [35]. Among these lipoprotein subunits, BamB is found in many bacteria species [35]. It adopts a unique β‐propeller‐like structure [36]. The side faced to the outer membrane is designated as the ‘upper side’, whereas the opposite side is designated as the ‘bottom side’. BamB directly interacts with BamA through loops in the ‘upper side’ [24]. BamB knockout impacts the biogenesis of a subtype of OMPs [37]. BamB might either regulate the function of the BAM complex or recruit client proteins [38], but how BamB behaves during the biogenesis of OMPs is unclear.

Periplasmic chaperones are also indispensable in the biogenesis of OMPs [20, 27, 39], although they are not conserved across species. Among the periplasmic chaperones, SurA is considered as the primary periplasmic chaperone [19, 20, 40], which has been demonstrated to directly interact with BamA [25, 41]. In addition, it binds both precursors (with a signal peptide at the N terminus) of OMPs and the mature unfolded OMPs, thereby making it possible to participate in not only the early stage (delivering nascent OMPs to BamA) but also the late stage (folding and membrane integration of OMPs) of the biogenesis of OMPs [20]. SurA has a core module consisting of the N domain, P1 domain and C domain, as well as an additional satellite P2 domain. It was reported that SurA interacted with BamA via the P2 domain, while interacting with the nascent OMPs with the N domain [25]. None of the chaperones, including SurA, is essential, although double knockout of these protein factors usually leads to the synthetic lethal phenotype, suggesting functional redundancy among these periplasmic chaperones [20]. Besides, the *bamB* knockout and *surA* mutation also resulted in the synthetic lethal phenotype [42], but the functional relationships between them were still less understood.

Studying the behavior of BamA is significant to uncover the mechanism for the biogenesis of OMPs because it is essential and conserved. However, to comprehensively understand the mechanism of the supercomplex in the biogenesis of OMPs, how the other components behave in the supercomplex and the crosstalk between components should be investigated. Therefore, in this work, the functional cooperation between BamA, BamB and SurA was studied; moreover, the association of SurA with the BAM complex in different conformations was predicted. First, photo‐cross‐linking experiments [25, 43, 44] were performed by introducing an unnatural amino acid [pBpa (*p*‐benzoyl‐l‐phenylalanine)] into specific residue positions of BamA and BamB to investigate the crosstalk between BamA/BamB and SurA in living cells. It was discovered that besides the periplasmic domains of BamA, the BamB subunit also directly interacted with SurA. Next, dual photo‐cross‐linking experiments revealed that solely one SurA molecule directly interacted with both BamA and BamB simultaneously, and a ternary complex composed of BamA, BamB and SurA was captured in living cells. In addition, the association of SurA with the BAM complex in either the ‘close’ or the ‘open’ conformation was predicted through the protein modeling. Finally, these theoretical models for the part of the supercomplex consisting of SurA and the BAM complex were used to interpret how they work together in the supercomplex during the biogenesis of OMPs and to preliminarily describe the entire process. The conservation and the diversity of the supercomplex were discussed.

## Results

### One SurA molecule bound to the POTRA1 and POTRA2 of the BamA subunit

**Fig. 1:**
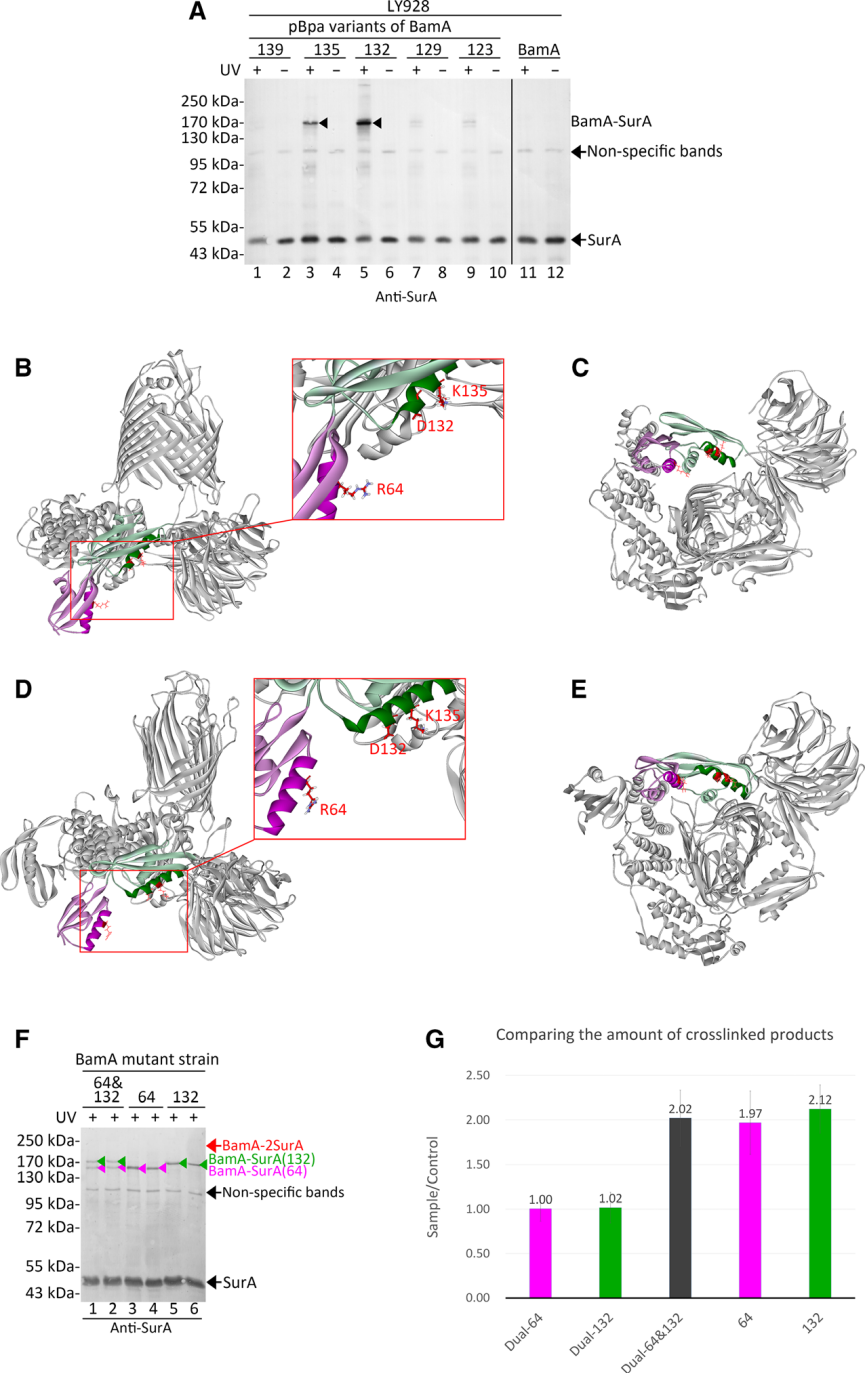
One SurA molecule bound to the second α‐helix of both the POTRA1 and POTRA2 domains of BamA. (A) Shown are immunoblotting results for the detection of the photo‐cross‐linked products of the indicated pBpa variants of BamA that were expressed in the LY928 strain, probed with antibodies against SurA. The cross‐linked BamA–SurA was indicated by the black arrowheads. Samples of cells expressing wild‐type BamA (with no pBpa incorporation) were analyzed as negative controls (lanes 11 and 12). Samples of cells without UV exposure were analyzed as negative controls (lanes 2, 4, 6, 8, 10 and 12) as well. (B–E) Positions of residues (colored red and shown as stick) intensively interacting with SurA were mapped and labeled in the experimentally determined structure of the BAM complex in either the ‘close’ (B and C, PDB: 5AYW) or the ‘open’ conformation (D and E, PDB: 5LJO ). The POTRA1 and POTRA2 domains of BamA were colored light pink and light green, respectively. The second α‐helix in the POTRA1 and POTRA2 domains was colored pink and green, respectively. (C and E) The bottom view of the BAM complex. (F) Shown are blotting results for the detection of the dual photo‐cross‐linked products in the indicated BamA mutant strains, which were constructed by replacing the wild‐type *bamA* gene with the *bamA* mutant gene encoding the indicated pBpa variants of BamA, probed with antibodies against SurA. ‘64 and 132’ (lanes 1 and 2) represented the LY928‐Avi‐BamA‐R64&D132pBpa strain in which pBpa was inserted in the residue positions 64 and 132 of BamA simultaneously. ‘64’ (lanes 3 and 4) represented the LY928‐Avi‐BamA‐R64pBpa strain in which pBpa was inserted in the residue position 64 of BamA. ‘132’ (lanes 5 and 6) represented the LY928‐Avi‐BamA‐D132pBpa strain in which pBpa was inserted in the residue position 132 of BamA. The cross‐linked BamA–SurA that was captured at the residue position 64 or 132 of BamA in each mutant strain was indicated by the pink or green arrowheads, respectively. The expected position of the dual photo‐cross‐linked product of BamA–2SurA was indicated on the right of the gel by the red arrow. (G) The immunoblotting results in (F) were analyzed with the ImageJ software, and the amount of the indicated cross‐linked products was illustrated in the chart. ‘Dual‐64’ and ‘Dual‐132’ represented the amount of cross‐linked products captured at the residue positions 64 (pink arrowhead) and 132 (green arrowhead) of BamA in the LY928‐Avi‐BamA‐R64&D132pBpa strain (lanes 1 and 2 in F). ‘Dual‐64&132’ represented the sum of the amount of the above two cross‐linked products. ‘64’ represented the amount of cross‐linked products captured at the residue position 64 of BamA (pink arrowhead) in the LY928‐Avi‐BamA‐R64pBpa strain (lanes 3 and 4 in F). ‘132’ represented the amount of cross‐linked products captured at the residue position 132 of BamA (green arrowhead) in the LY928‐Avi‐BamA‐D132pBpa strain (lanes 5 and 6 in F). The *n* value, which represented the number of biologically independent replicates, was 2. The error represented the standard deviation. All protein samples in (A) and (F) were resolved by SDS/PAGE before being subjected to the blotting analysis. Residue positions for BamA were numbered by including the signal peptides. Positions of protein monomers, nonspecific bands and photo‐cross‐linked products were indicated on the right of the gel. Positions of the molecular mass markers were on the left.

SurA has been demonstrated to be the primary chaperone [19, 20, 40] and an important component of the supercomplex [25]. Therefore, in this work, the crosstalk between BAM subunits and SurA was studied to investigate the detailed mechanism. Previous study indicated that the second α‐helix of the POTRA1 domain of BamA directly interacted with SurA, and the interaction was most intense at Arg64 of BamA [41]. Similar results were observed in this work through photo‐cross‐linking experiments (Fig. S1); moreover, a new binding region was identified (Fig. 1A). The unnatural amino acids (pBpa) were inserted in specific residue positions of BamA through site‐specific mutagenesis. In the LY928 (Table S1) *E. coli* cells, these pBpa variants of BamA and the wild‐type BamA protein (as the negative control, lanes 11 and 12 in Fig. 1A) were expressed from low‐copy plasmids under the control of its natural promoters, respectively (Table S2). A streptavidin tag (Avi‐tag) was linked to the N terminus of BamA and BamA variants (Table S2) for the blotting analysis and purification. The cells in the mid‐log phase were irradiated under the UV light for 10 min and harvested before being subjected to the immunoblotting analysis (lanes 1 and 3 in Fig. S1; lanes 1, 3, 5, 7, 9 and 11 in Fig. 1A). Samples of cells without the UV exposure were also prepared as negative controls (lanes 2 and 4 in Fig. S1; lanes 2, 4, 6, 8, 10 and 12 in Fig. 1A). Probed with antibodies against SurA (Fig. S1), the cross‐linked BamA (~95 kDa) and SurA (~47 kDa) with an apparent molecular mass of ~142 kDa were detected in the BamA‐D58pBpa (very weak, lane 1) and the BamA‐R64pBpa variants (intensive, lane 3), indicating that residue 64 of BamA directly interacted with SurA intensively. In addition, I have designed and constructed various pBpa variants of BamA and identified a novel binding site (Lys135) for SurA in the POTRA2 domain of BamA [25]. In this work, it was further revealed that besides the BamA‐K135pBpa variant (intensive, lane 3), the cross‐linked products of BamA–SurA with an apparent molecular mass of ~170 kDa were also detected in the BamA‐D132pBpa variant (intensive, lane 5), as well as the BamA‐T129pBpa and BamA‐E123pBpa variants (very weak, lanes 7 and 9), probed with antibodies against SurA (Fig. 1A). The apparent molecular mass of cross‐linked products captured in the POTRA2 domain (~170 kDa; lanes 3 and 5 in Fig. 1A) was slightly higher than that in the POTRA1 domain (~ 142 kDa; lane 3 in Fig. S1) because different types of cross‐linked products may be obtained when pBpa was inserted in different residue positions. Different types of cross‐linked products have different migration speeds in the gel, resulting in different apparent molecular masses. Then plasmids expressing the BamA‐R64pBpa and BamA‐D132pBpa variants were transformed into the *surA* knockout strain (the LY928‐∆s*urA* strain in Table S1). In the LY928‐∆*surA* strain, the protein bands of ~142 or ~170 kDa disappeared (lanes 5 and 6 in Fig. S1), further confirming that these protein bands detected by antibodies against SurA were the cross‐linked BamA and SurA. Besides, the cross‐linked products could be purified on the streptavidin‐affinity column through the Avi‐tag linked to the N terminus of BamA and were sent for mass spectrometry analysis. SurA was identified in the UV‐exposed sample, but not the negative control sample (Table S3).

Herein the residues that intensively interacted with SurA were mapped and labeled in the structure of the BAM complex adopting either the ‘close’ [Fig. 1B,C, Protein Data Bank (PDB): 5AYW] or the ‘open’ conformation (Fig. 1D,E, PDB: 5LJO). Besides the second α‐helix of POTRA1, the second α‐helix of POTRA2 was a new binding region for SurA. The labeled residues are exposed to the periplasm and thus are proper for interacting with SurA.

Because two binding regions (Fig. 1B–E) for SurA at the POTRA1 and the POTRA2 domains of BamA were identified, respectively, one may wonder whether there are one or two SurA molecules that directly interacted with BamA at these two regions. To interpret this issue, pBpa was inserted in the residue positions 64 and 132 of BamA simultaneously to perform dual photo‐cross‐linking experiments. The wild‐type *bamA* gene was replaced by the *bamA* mutant gene encoding BamA‐R64pBpa, BamA‐D132pBpa or BamA‐R64&D132pBpa variants in the LY928 strain (Table S1). The growth of these BamA mutant strains was not impacted, indicating that these mutations did not severely affect the biogenesis and function of BamA. The photo‐cross‐linking experiments with these BamA mutant strains were repeated twice (Fig. 1F). Probed with antibodies against SurA, two cross‐linked products were simultaneously detected (lanes 1 and 2 in Fig. 1F) in the LY928‐Avi‐BamA‐R64&D132pBpa strain. These two cross‐linked products were respectively identical with the cross‐linked BamA‐SurA (Fig. 1F) captured in the LY928‐Avi‐BamA‐R64pBpa (lanes 3 and 4) or LY928‐Avi‐BamA‐D132pBpa strain (lanes 5 and 6). The immunoblotting results in Fig. 1F were then analyzed with the ImageJ software, using the indicated nonspecific bands as the internal control. The analysis results (Table S4) were displayed in the chart (Fig. 1G), which demonstrated that the sum (2.02) of the amount of the two cross‐linked products (1.00 and 1.02) in the LY928‐Avi‐BamA‐R64&D132pBpa strain was similar to the amount of the cross‐linked BamA‐SurA in either the LY928‐Avi‐BamA‐R64pBpa (1.97) or the LY928‐Avi‐BamA‐D132pBpa strain (2.12). If two SurA molecules bind to the POTRA1 and POTRA2 domains of BamA, respectively, a new cross‐linked product of BamA‐2SurA with higher molecular mass (the molecular mass of BamA plus the double of the molecular mass of SurA, ~189 kDa) would be captured. The apparent molecular mass of BamA‐SurA captured at POTRA2 is higher than the calculated one, so the apparent molecular mass of BamA‐2SurA may be a little higher than the calculated one as well. However, no new cross‐linked products around ~189 kDa were detected in Fig. 1F, indicating that solely one SurA molecule directly interacted with BamA, although SurA may function as a dimer.

### SurA directly interacts with the BamB subunit

Photo‐cross‐linking results in Fig. 1A demonstrated that the Lys135 residue of BamA directly interacted with SurA, whereas in the experimentally determined structure of the BAM complex in either the ‘close’ (Fig. S2A,B; PDB: 5AYW) or the ‘open’ conformation (Fig. S2C,D; PDB: 5LJO), the Lys135 residue was in the binding interface between BamA and BamB (Fig. S2B,D). It was suggested that the binding regions of SurA and BamB in BamA are quite close and even partially overlapped around residue 135 of BamA, but no interaction between SurA and BamB has been reported in living cells before. Therefore, I performed photo‐cross‐linking experiments by introducing pBpa in the BamB subunit to investigate whether and how BamB interacts with SurA.

**Fig. 2:**
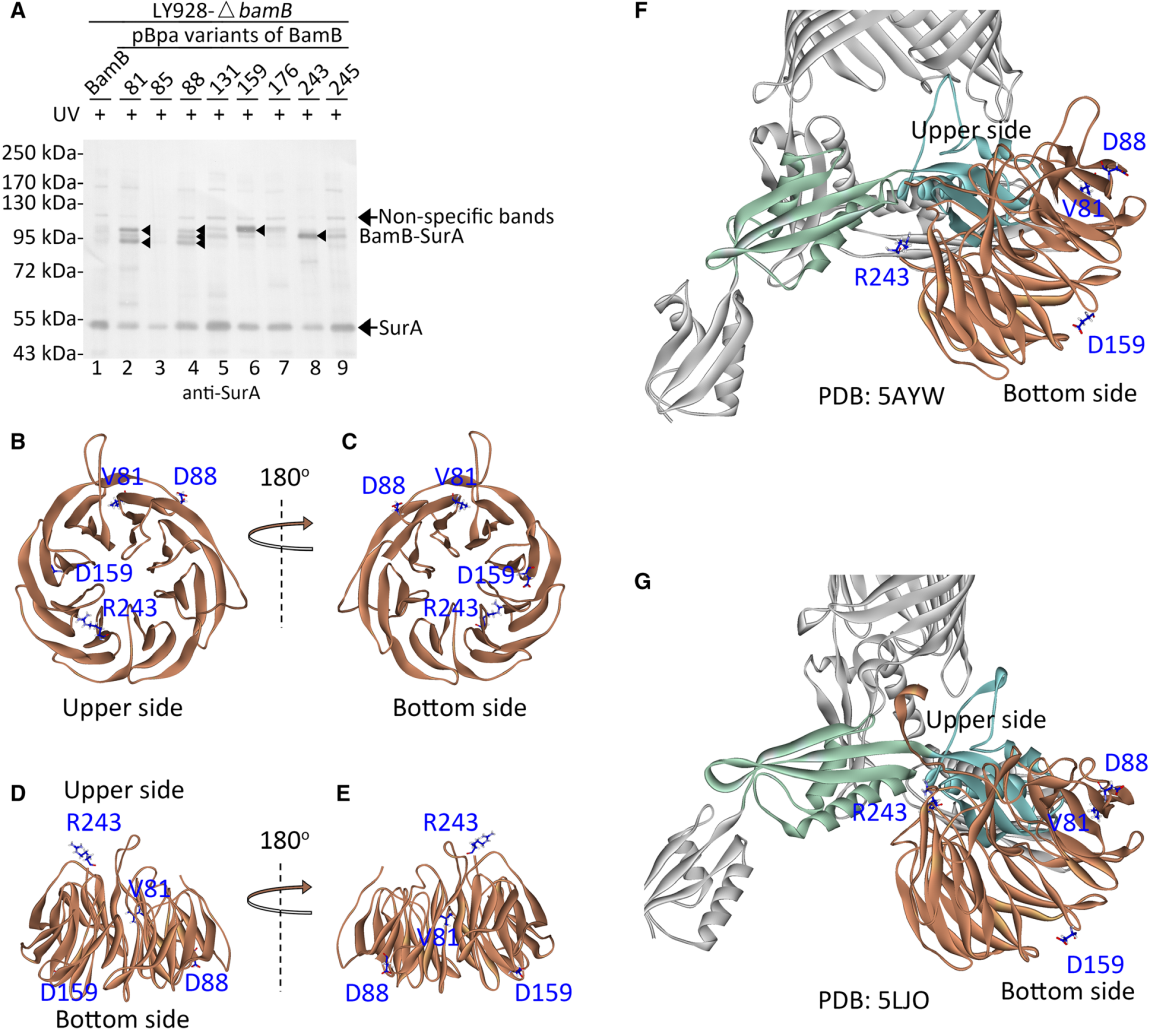
BamB directly interacted with SurA in living cells. (A) Shown are immunoblotting results for the detection of the photo‐cross‐linked products of the indicated pBpa variants of BamB that were expressed in the LY928‐∆*bamB* strain, probed with antibodies against SurA. The cross‐linked BamB–SurA was indicated by the black arrowheads. Samples of cells expressing wild‐type BamB (with no pBpa incorporation) were analyzed as the negative control (lane 1). All protein samples were resolved by SDS/PAGE before being subjected to the immunoblotting analysis. Residue positions for BamB were numbered by including the signal peptides. Positions of protein monomers, nonspecific bands and photo‐cross‐linked products were indicated on the right of the gel. Positions of the molecular mass markers were on the left. (B–E) Positions of residues (colored blue and shown as stick) intensively interacting with SurA were mapped and labeled in the experimentally determined structure of BamB (PDB: 5AYW, chain B, light brown) displayed from different orientations. (F, G) Positions of residues (colored blue and shown as stick) intensively interacting with SurA were mapped and labeled in the experimentally determined structure of the BAM complex in either the ‘close’ (PDB: 5AYW) or the ‘open’ conformation (PDB: 5LJO). Solely the BamA and BamB subunit were shown. BamB interacted with the POTRA2 (light green) and POTRA3 (light cyan) domains of BamA through loops in the ‘upper side’.

The pBpa variants of BamB and the wild‐type BamB protein (as the negative control, lane 1 in Fig. 2A) were expressed from low‐copy plasmids under the control of its natural promoter (Table S2) in the LY928‐∆*bamB* cells (Table S1). The cells in the mid‐log phase were irradiated under UV for 10 min and harvested before being subjected to the immunoblotting analysis. Probed with antibodies against SurA (Fig. 2A), cross‐linked BamB and SurA were intensively detected in the BamB‐V81pBpa (lane 2), BamB‐D88pBpa (lane 4), BamB‐D159pBpa (lane 6) and BamB‐R243pBpa (lane 8) variants. Such protein bands of the cross‐linked products were obviously detected (lanes 2, 4, 6 and 8 in Fig. 2A) when the pBpa variants of BamB were expressed in the LY928‐∆*bamB* strain, but were not detected (lanes 7–10 in Fig. S1) when they were expressed in the LY928‐∆*surA* stain (Table S1), further confirming that these protein bands represented the cross‐linked BamB and SurA. The apparent molecular mass (between ~85 and ~100 kDa) of these products are around the sum (~87 kDa) of the molecular mass of BamB (~40 kDa) and SurA (~47 kDa) but with slight differences, because these cross‐linked products of BamB‐SurA were captured between different residues of BamB and SurA. These different types of cross‐linked products may have different migration speeds in the gel, and thus their apparent molecular mass was different. These results demonstrated direct interactions between BamB and SurA. The identified binding site residues were scattered in different regions of BamB (PDB: 5AYW, chain B, Fig. 2B–E) and could not be clustered together, suggesting that there may be multiple binding interfaces between BamB and SurA. The residue positions 159 and 243 were respectively located at the ‘bottom side’ and the ‘upper side’ of BamB. The loops at the ‘upper side’ of BamB interacted with the POTRA2 and POTRA3 of BamA, when the BAM complex was in either the ‘close’ (Fig. 2F, PDB: 5AYW) or the ‘open’ conformation (Fig. 2G, PDB: 5LJO); thus, residue 243 was close to BamA, whereas residue 159 was at the opposite side. In addition, more than one cross‐linked product was detected in the BamB‐V81Bpa and BamB‐D88pBpa variants, suggesting that the interactions between BamB and SurA might be dynamic, or such interactions may occur in some intermediate states.

### A ternary BamA–BamB–SurA complex was captured in living cells

**Fig. 3:**
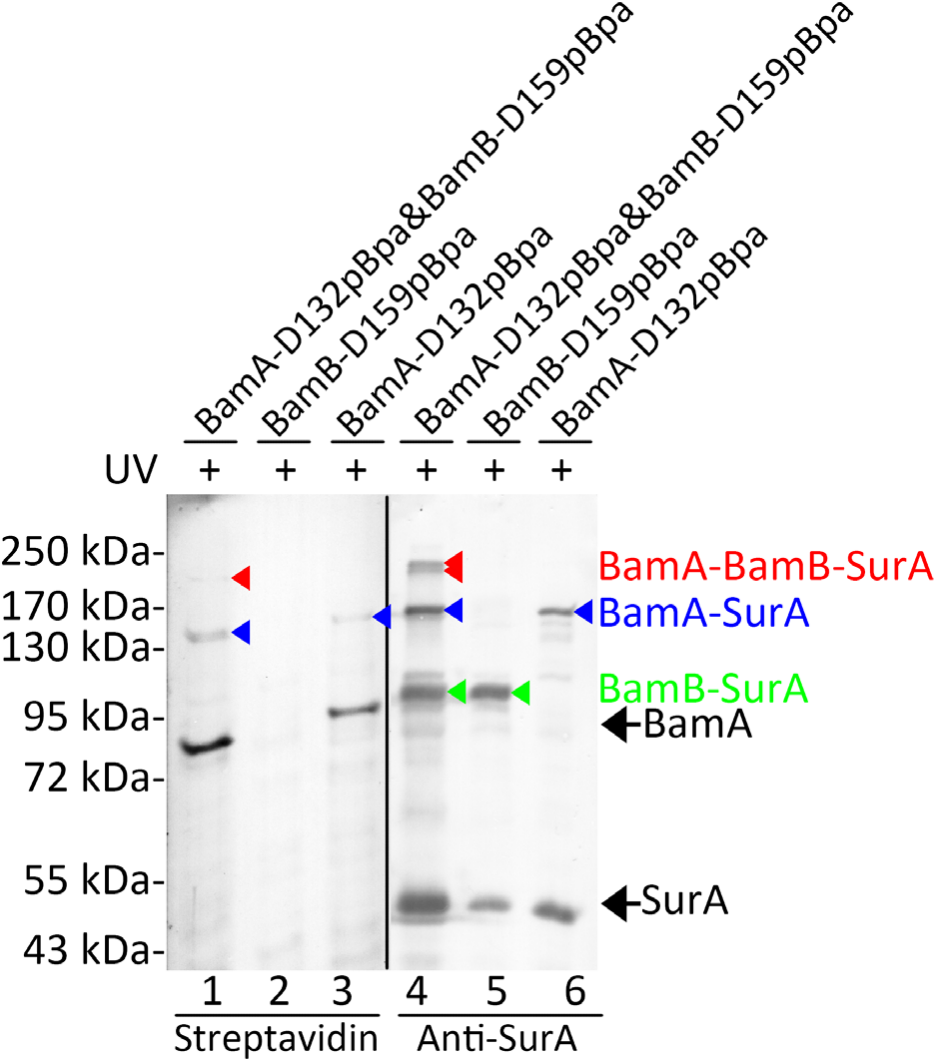
A ternary BamA–BamB–SurA complex was captured in living cells. Blotting results for the detection of photo‐cross‐linked products formed in the LY928 cells expressing the indicated pBpa variants of BamA and/or BamB probed with streptavidin‐AP conjugate against the Avi‐tag linked to BamA (lanes 1, 2 and 3) or with antibodies against SurA (lanes 4, 5 and 6). Protein samples were resolved by SDS/PAGE before being subjected to the blotting analysis. The cross‐linked products of BamA‐SurA, BamB‐SurA and BamA‐BamB‐SurA were indicated by the blue, green and red arrowheads, respectively. Positions for BamA, SurA, BamA‐SurA, BamB‐SurA or BamA‐BamB‐SurA are indicated on the right, and positions for the molecular mass markers are on the left.

The earlier photo‐cross‐linking results demonstrated that SurA interacted with both the BamA and BamB subunits. One may wonder whether one SurA molecule directly interacted with both BamA and BamB, or two SurA molecules (maybe as a dimer) directly interacted with BamA and BamB, respectively. Therefore, dual photo‐cross‐linking experiments were designed to investigate this issue. Because solely one SurA molecule directly interacted with BamA at residue positions 64, 132 and 135, pBpa could be inserted in any of them for the dual photo‐cross‐linking experiments. The wild‐type *bamA* gene was replaced by the *bamA* mutant gene encoding the BamA‐D132pBpa variant through recombination in the LY928 strain (Table S1). The efficiency for capturing the ternary complex through dual photo‐cross‐linking experiments was relatively low; thus, the residue position 159 of BamB that interacted with SurA most intensively was favorable. In the LY928‐Avi‐BamA‐D132pBpa strain, the BamB‐D159pBpa variant was expressed from low‐copy plasmids under the control of its natural promoter to perform dual photo‐cross‐linking experiments (lanes 1 and 4 in Fig. 3). The LY928 strain transformed with plasmids expressing the BamB‐D159pBpa variant (lanes 2 and 5 in Fig. 3) and the LY928‐Avi‐BamA‐D132pBpa strain transformed with plasmids expressing the wild‐type BamB (lanes 3 and 6 in Fig. 3) were used as negative controls. Probed with the streptavidin‐AP (alkaline phosphatase) conjugate (lanes 1–3), as well as antibodies against SurA (lanes 4–6), photo‐cross‐linked products (lanes 1 and 4 in Fig. 3) with the apparent molecular mass of ~210 kDa were detected. The apparent molecular mass of BamA–BamB–SurA almost equaled the sum (~210 kDa) of the molecular mass of BamA–SurA (~170 kDa) and the molecular mass of BamB (~40 kDa), which is slightly higher than the calculated one (~182 kDa). The cross‐linked products of BamA‐BamB‐SurA were not obviously detected when they were probed with antibodies against BamB (data not shown) due to the relatively low sensitivity of antibodies against BamB and the relatively low efficiency of the dual cross‐linking. This product was not detected (Fig. 3) in the control samples when solely BamB‐D159pBpa (lanes 2 and 5) or BamA‐D132pBpa (lanes 3 and 6) variants were expressed in the cells. These results demonstrated a complex composed of BamA, BamB and SurA in living cells. Considering the size and geometry of the BAM complex and SurA, it was possible that one SurA molecule simultaneously interacted with both BamA and BamB when it was associated with the BAM complex. Moreover, at least two cross‐linked products with apparent molecular mass around ~210 kDa were clearly detected with antibodies against SurA, demonstrating that different forms of cross‐linked BamA‐BamB‐SurA were captured. It was suggested that the SurA–BAM complex may have different conformations.

### Theoretical structures for the SurA–BAM complex in different conformations were constructed using ZDOCK

BamA, BamB and SurA were functional partners in the supercomplex [25]. The structures for the BAM complex have been experimentally determined, but how SurA was associated with the BAM complex and how they cooperated in the biogenesis of OMPs were less understood. Herein, through protein modeling, the binding of SurA with the BAM complex was studied. Because some residues in the experimentally determined structures for SurA were missing, the homology model for SurA was built according to the experimentally determined structures (PDB: 1M5Y and 2PV1). The four chains of 1M5Y (1M5Y _a, _b, _c, _d) and 2PV1 were input as templates to generate homology models for SurA in *E. coli*. The structural similarities of these templates were compared. The main‐chain RMSD and the number of overlapping residues were listed in Table S5. The related properties of the generated models were listed in Table S6. The model with the lowest PDF Total Energy (SurA_01, Table S6 and Fig. S3A) was optimal and used in the following modeling processes. The optimal model of SurA was verified with the Ramachandran plot (Fig. S3B), in which almost all the residues except for six were in the favorable regions. The six unfavorable residues were located in the loops and β‐strands (Fig. S3C). The results of Profiles‐3D were displayed in Fig. S3D. The Verify Score of SurA_01 was 167.31 (Table S7), which was much higher than the expected low score (82.92) and approached the expected high score (184.27). The results of verification (Fig. S3B,D and Table S7) demonstrated that the structure of SurA was mostly correctly built. The structural similarity between SurA_01 and the templates was shown in Fig. S3E. The main‐chain RMSD and the number of overlapping residues were listed in Table S5.

SurA_01 (the ligand) was then docked to the experimentally determined structure of the BAM complex (the receptor) in either the ‘close’ (PDB: 5AYW) or the ‘open’ conformation (PDB: 5LJO) using the ZDOCK algorithm. The receptor‐blocked residues were those in the C‐terminal region of BamA, which was embedded in the outer membrane. The obtained poses were clustered according to the position of the ligand. Poses were filtered by setting specific binding site residues according to the experimental information obtained previously [25, 41] and in this work (Figs 1A,F,2A and 3). First, BamA interacted intensively with SurA at the residue positions 64, 132 and 135. Second, BamB interacted intensively with SurA at the residue positions 159 and 243. Third, SurA mainly interacted with BamA through the satellite P2 domain indicated in Fig. S3A. With these criteria, proper models were filtered. All poses were displayed with the 3D plot (Fig. S4A,B) to indicate ones with a high ZDOCK score and a high density. Properties for the selected models with a high ZDOCK score and a high density were listed in Tables S8 and S9.

**Fig. 4:**
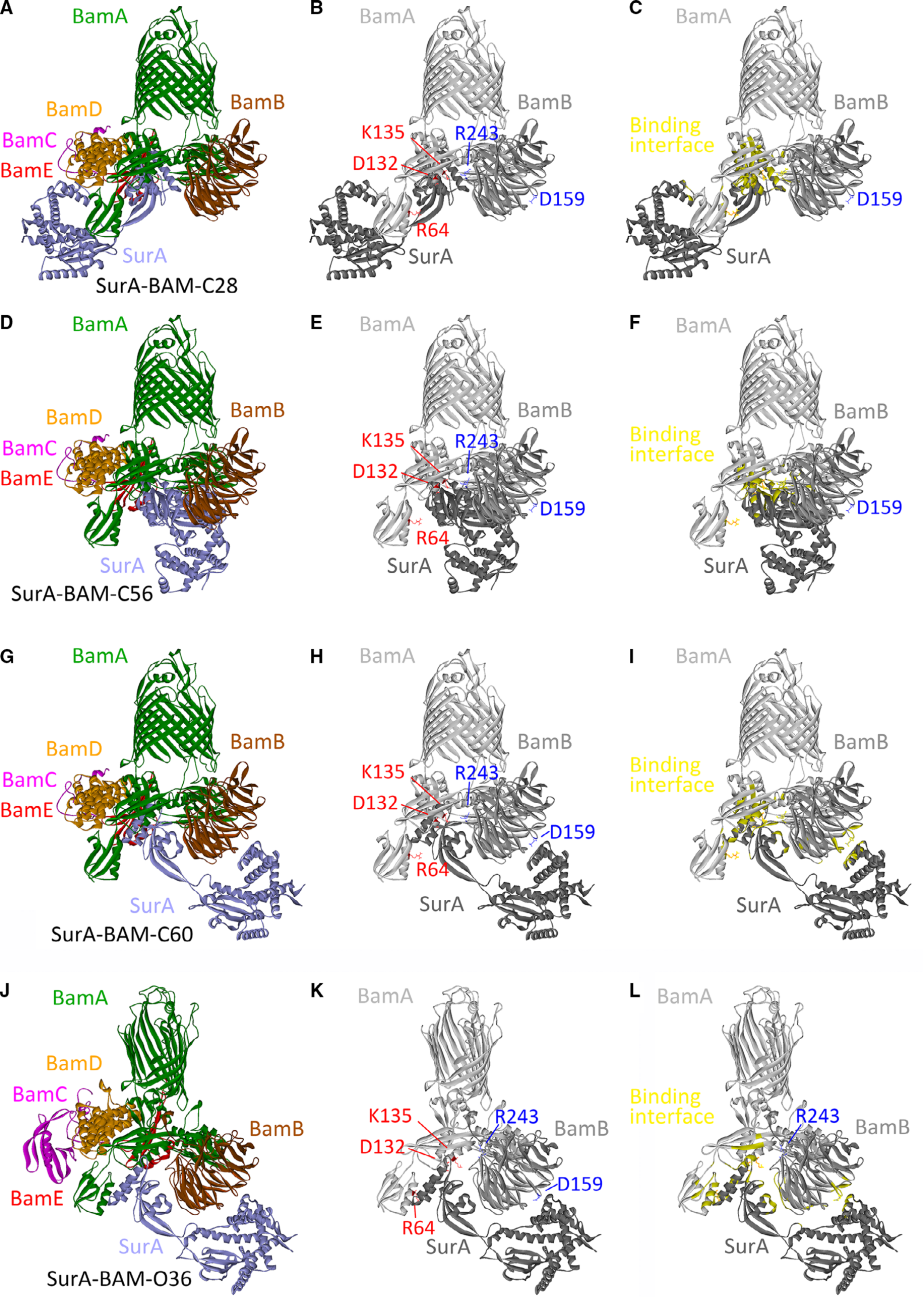
Theoretical structures for the SurA–BAM complex were constructed. (A, D, G, J) The model of SurA was docked to the BAM complex in either the ‘close’ (A, D, G; PDB: 5AYW) or the ‘open’ conformation (J; PDB: 5LJO). The subunits of the BAM complex were colored green (BamA), brown (BamB), pink (BamC), orange (BamD) and red (BamE); SurA was colored light purple. (B, E, H, K) Displayed are positions of the residues 64, 132 and 135 of BamA, which were colored red and shown as stick, as well as positions of the residues 159 and 243 of BamB, which were colored blue and shown as stick. Solely the BamA (colored light gray) and BamB (colored gray) subunits of the BAM complex, as well as SurA (colored dark gray), were shown. The BAM complex was in the ‘close’ conformation in (B), (E) and (H), but in the ‘open’ conformation in (K). (C, F, I, L) The binding interface between SurA and the BAM complex was highlighted in yellow. Solely the BamA and BamB subunits of the BAM complex, as well as SurA, were displayed. BamA, BamB and SurA were colored light gray, gray and dark gray, respectively. The BAM complex was in the ‘close’ conformation in (C), (F), and (I), while in the ‘open’ conformation in (L). The experimentally identified binding site residues, including the residues 64, 132 and 135 of BamA, as well as the residues 159 and 243 of BamB, which are located outside the binding interfaces in the models, were labeled and shown as stick.

SurA_01 could be docked to the BAM complex in either the ‘close’ or the ‘open’ conformation. The representative models meeting with the earlier criteria were shown in Fig. 4. When the BAM complex was in the ‘close’ conformation, the SurA–BAM complex (‘close’) could adopt different conformations, in which SurA oriented to at least three directions (Fig. 4A,D,G). However, when the BAM complex was in the ‘open’ conformation, the SurA–BAM complex (‘open’) mainly preferred one conformation (Fig. 4J). These models were designated as SurA–BAM–C28 (Fig. 4A), SurA–BAM–C56 (Fig. 4D), SurA–BAM–C60 (Fig. 4G) and SurA–BAM–O36 (Fig. 4J). The experimentally identified binding site residues were labeled in these four models (Fig. 4B,E,H,K). The binding interfaces between BamA and SurA, as well as between BamB and SurA, were indicated; meanwhile, the experimentally identified binding site residues that were located outside the binding interfaces in the models were labeled (Fig. 4C,F,I,L). Consistent with the experimental results, SurA mainly bound to the POTRA1 and POTRA2 domains of BamA through its P2 domain in all these models (Tables S8 and S9), in similar manners with those shown in Fig. 4. The second α‐helices of the POTRA1 and POTRA2 domains were involved in the interaction. Residues 64, 132 and 135 of BamA directly interacted with SurA in all of these models (Tables S8 and S9). When the SurA–BAM complex was in the ‘close’ conformation, residue 243 of BamB was in the binding interface between BamB and SurA in all the filter models except for pose 49 (Table S8; Fig. 4C,F,I), but residue 159 of BamB was solely in the binding interface between BamB and SurA in poses 1, 49, 53 and 60 (Table S8 and Fig. 4I). Moreover, in models (poses 1, 53 and 60 in Table S8) such as SurA–BAM–C60 (Fig. 4G), both residues 159 and 243 of BamB were in the binding interface between BamB and SurA (Fig. 4I). When the BAM complex was in the ‘open’ conformation, residue 159 rather than residue 243 of BamB was in the binding interface between BamB and SurA (Table S9 and Fig. 4L). Taken together, the SurA–BAM complex could adopt different conformations and may undergo conformational change during functioning.

### The BamB subunit could bind a new region of BamA during functioning, suggesting new conformations for the SurA–BAM complex

**Fig. 5:**
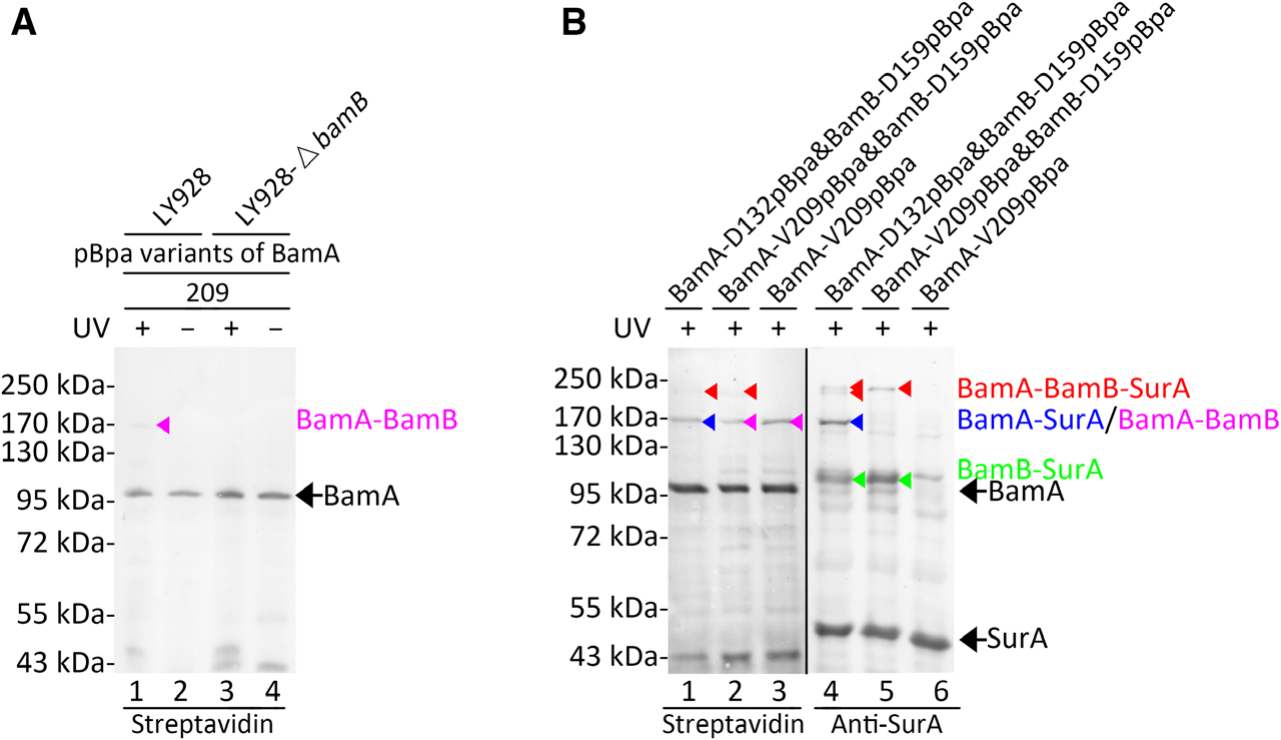
A new binding site for BamB was identified in BamA; meanwhile, the BamA–BamB–SurA complex was captured at this residue position in living cells. (A) Shown are blotting results for the detection of the photo‐cross‐linked products of the indicated pBpa variants of BamA that were expressed in the LY928 (lanes 1 and 2) or the LY928‐∆*bamB* strain (lanes 3 and 4), probed with streptavidin‐AP conjugate against the Avi‐tag linked to BamA. The cross‐linked BamA–BamB was indicated by the pink arrowhead. Samples of cells without the UV exposure were analyzed as negative controls (lanes 2 and 4). (B) Shown are blotting results for the detection of photo‐cross‐linked products formed in the LY928 cells expressing the indicated pBpa variants of BamA and/or BamB, probed with streptavidin‐AP conjugate against the Avi‐tag linked to BamA (lanes 1, 2 and 3) or with antibodies against SurA (lanes 4, 5 and 6). The dual photo‐cross‐linking experiments in Fig. 3 (lanes 1 and 4) were repeated here (lanes 2 and 5) for comparison. The cross‐linked products of BamA–SurA, BamA–BamB, BamB–SurA and BamA–BamB–SurA were indicated by the blue, pink, green and red arrowheads, respectively. Protein samples were resolved by SDS/PAGE before being subjected to the blotting analysis. Positions for BamA, SurA and the cross‐linked products were indicated on the right, and positions for the molecular mass markers were on the left.

The SurA–BAM complex may adopt new conformations in which BamB moved to a new position, because a new binding site for BamB was identified in BamA in this work through photo‐cross‐linking experiments. A photo‐cross‐linked product was detected in the BamA‐V209pBpa variant (lane 1) by the blotting analysis, probed with the streptavidin‐AP conjugate (Fig. 5A). It was further confirmed by the mass spectrometry analysis (Table S10). BamB was detected in the cross‐linked samples (repeated twice), but not in the control sample. Moreover, this cross‐linked product could not be detected when the BamA‐V209pBpa variant was expressed in the LY928‐∆*bamb* strain (lane 3 in Fig. 5A). These results indicated that the Val209 residue of BamA directly interacted with BamB. However, the Val209 residue of BamA was not inside the binding interface between BamA and BamB that was identified in the experimentally determined structure of the BAM complex adopting either the ‘close’ (PDB: 5AYW) or the ‘open’ conformation (PDB: 5LJO), suggesting that the binding region of BamB could be changed during functioning in the supercomplex. BamB could be at new positions that were slightly different from that in the structure of the isolated BAM complex. Indeed, dual photo‐cross‐linking experiments, which were performed by expressing the BamB‐D159pBpa variant (Table S2) in the LY928‐Avi‐BamA‐V209pBpa strain (Table S1), captured a ternary BamA–BamB–SurA complex (~210 kDa) in living cells (lanes 2 and 5 in Fig. 5B). Solely one dual cross‐linked product of about ~210 kDa was detected here (lanes 2 and 5 in Fig. 5B), suggesting that apparently one form of the BamA–BamB–SurA complex was captured. But two dual photo‐cross‐linked products (~210 kDa) were obtained when BamA‐D132pBpa and BamB‐D159pBpa variants were expressed (lanes 1 and 4). These two forms of the BamA–BamB–SurA complex may result from different positions of BamB in the complex. No cross‐linked product around ~210 kDa was detected in the negative control expressing the wild‐type BamB in the LY928‐Avi‐BamA‐V209pBpa strain (lanes 3 and 6).

**Fig. 6:**
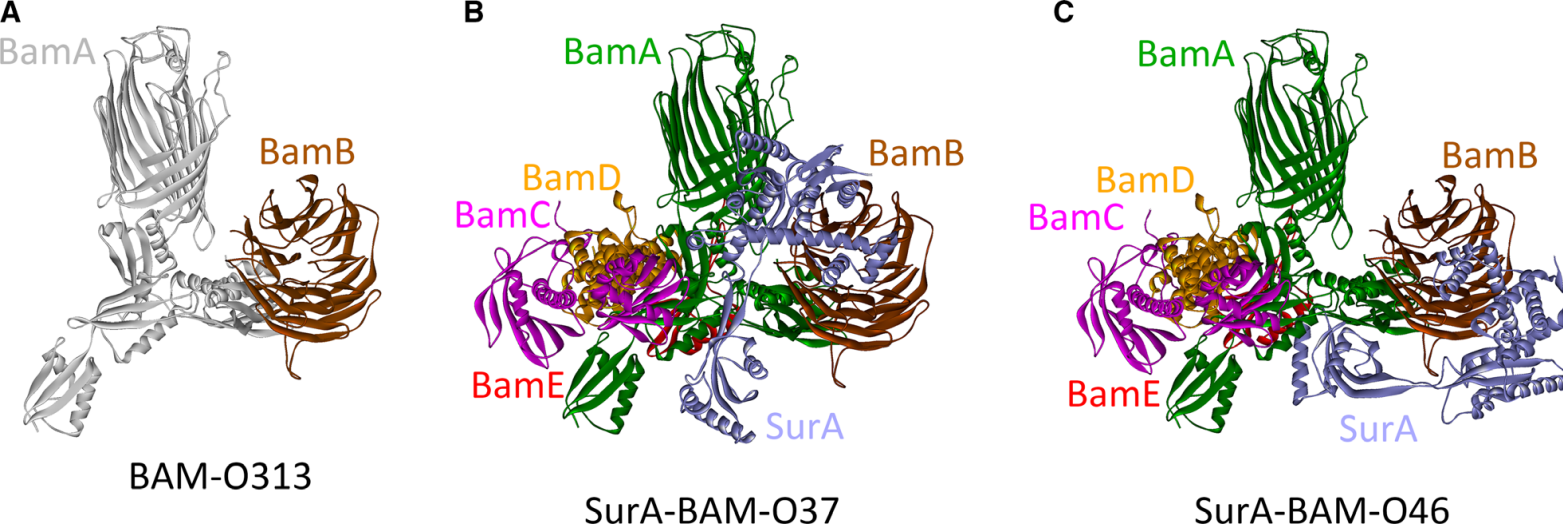
Theoretical structures for the SurA–BAM complex in which BamB bound to the new region was constructed using ZDOCK. (A) BamB was docked to the BamACDE complex in the ‘open’ conformation (PDB: 5EKQ). Solely the BamA and BamB subunits were displayed. BamA was colored gray, and BamB was colored brown. (B, C) The model of SurA was docked to the BAM–O313 (A). Shown are two representative models, SurA–BAM–O37 and SurA–BAM–O46. The subunits of the BAM complex were colored green (BamA), brown (BamB), pink (BamC), orange (BamD) and red (BamE); SurA was colored light purple.

The earlier results suggested new conformations of the SurA–BAM complex; thus, protein modeling was used to construct theoretical structures for the SurA–BAM complex in which BamB bound to the new region. The structure for the BamACDE complex in which BamA adopted the ‘open’ conformation (PDB: 5EKQ) has been experimentally determined, but no such structure has been reported when BamA adopted the ‘close’ conformation. Thus, BamB was tried to be docked to the BamACDE complex in the ‘open’ conformation (PDB: 5EKQ). The residue 209 of BamA that interacted with BamB was located in a long loop of POTRA3; thus, proper poses were filtered according to the following criteria. First, BamB still mainly bound to the POTRA2 and POTRA3 domains of BamA. Its ‘upper side’ was still faced to BamA. Second, binding site residues were set to be those around residue 209. Filtered poses were displayed in Fig. S4C. Properties for the displayed model were listed in Table S11. The representative model was shown in Fig. 6A, designated BAM‐O313. It should be pointed out that the structures of the BamACDE complex may not be completely identical with that in the native structure of the BAM complex in which BamB bound to new regions, but they would not be quite different from that, because even losing BamB did not severely change the structures of the complex. Consequently, SurA was docked to the theoretical structure of the BAM complex in a new conformation (Fig. 6A). Similar criteria used in constructing theoretical structures in Fig. 4 were applied here, and the models for the SurA–BAM complex in new conformations were obtained. The filtered poses were displayed in Fig. S4D. Representative models for the SurA–BAM complex in new conformations were shown in Fig. 6B,C. In the new models, residue 64 of BamA did not interact with SurA, inferring that additional components in the supercomplex may be required to stabilize the SurA–BAM complex in the new conformations. Properties for the filtered models were listed in Table S12. The distances between SurA and the indicated residues of BamA were listed as well (Table S12). In addition, without BamB, SurA could still be docked to the BamACDE complex, but the SurA–BamACDE complex could adopt multiple conformations (Fig. S5). These results indicated that BamB could stabilize the SurA–BAM complex in particular conformations by regulating the interaction between BamA and SurA.

## Discussion

In this article, first, mainly through photo‐cross‐linking experiments, it was revealed that one SurA molecule directly interacted with the POTRA1 and POTRA2 domains of BamA (Figs 1A,F and S1). Second, photo‐cross‐linking experiments demonstrated that BamB directly interacted with SurA (Fig. 2A). Third, a ternary BamA–BamB–SurA complex was captured in living cells (Figs 3 and 5B). Fourth, through protein modeling, theoretical structures for the part of the supercomplex consisting of SurA and the BAM complex in different conformations were constructed (Figs 4 and 6).

The theoretical structures in this work were constructed mainly according to the cross‐linking results. When pBpa was incorporated in BamA, the photo‐cross‐linking experiments could demonstrate that R64, D132 and K135 residues of BamA interacted with SurA, but it was hard to determine to which residues of SurA they interacted. But Lys residues of SurA possibly interacted at close vicinity to R64 and Lys residues of BamA, because DSP (dithiobis(succinimidylpropionate))‐mediated BamA and SurA cross‐linking, as well as SPDP (N‐succinimidyl 3‐(2‐pyridyldithio) propionate)‐mediated BamA‐R64C mutant and SurA cross‐linking, have been reported [41]. DSP mediates amine‐to‐amine cross‐linking with a 12‐Å spacer arm length. SPDP mediates amine‐to‐sulfhydryl cross‐linking with a 6.8‐Å spacer arm length. So, Lys residues of SurA within the distance of 6.8 Å to R64 or of 12 Å to K135 residue of BamA were found in the theoretical structures. In almost all of the models listed in Tables S8, S9 and S12, one or more Lys residues of SurA were located in the vicinity (<12 Å) of K135 of BamA, and in models such as poses 1, 3, 19 and 60 in Table S8, as well as pose 36 in Table S9, Lys residues of SurA were also found in the vicinity of R64 (<6.8 Å) of BamA. The conservation of experimentally identified binding site residues in BamA and BamB that directly interacted with SurA were analyzed. The sequences of BamA and BamB proteins with relatively high identity (40%) to the sequences of *E. coli* BamA and BamB were searched in the Uniref90 database and aligned. Among these BamA proteins, the R64 residue of BamA was conserved and the K135 residue was relatively conserved. The residue 135 interacted with SurA in all of the filtered models. The residue 64 of BamA directly interacted with SurA in almost all of the filtered models except for those in Table S12. Maybe the interaction between R64 of BamA and SurA could be stabilized by the other components of the supercomplex (e.g., PpiD, inner membrane translocon, etc.) when BamB moved to the new binding regions. Among these BamB proteins, the residues 159 and 243 were conserved. Residues of SurA that directly interacted with BamA have been demonstrated in previous research [25]. In all of the displayed models (Figs 4 and 6) except for SurA‐BAM‐56, more than one of these residues could be found in the binding interface between BamA and SurA. Almost all of these residues were scattered in the P2 domain of SurA but oriented to different directions, suggesting multiple binding interfaces between BamA and SurA. Among these residues, the residues 311, 333 and 349 were conserved among SurA proteins with relatively high sequence identity (40%) to the *E. coli* SurA. The conservation of binding site residues inferred their importance for the protein interaction perhaps not only in *E. coli* but also in other species.

**Fig. 7:**
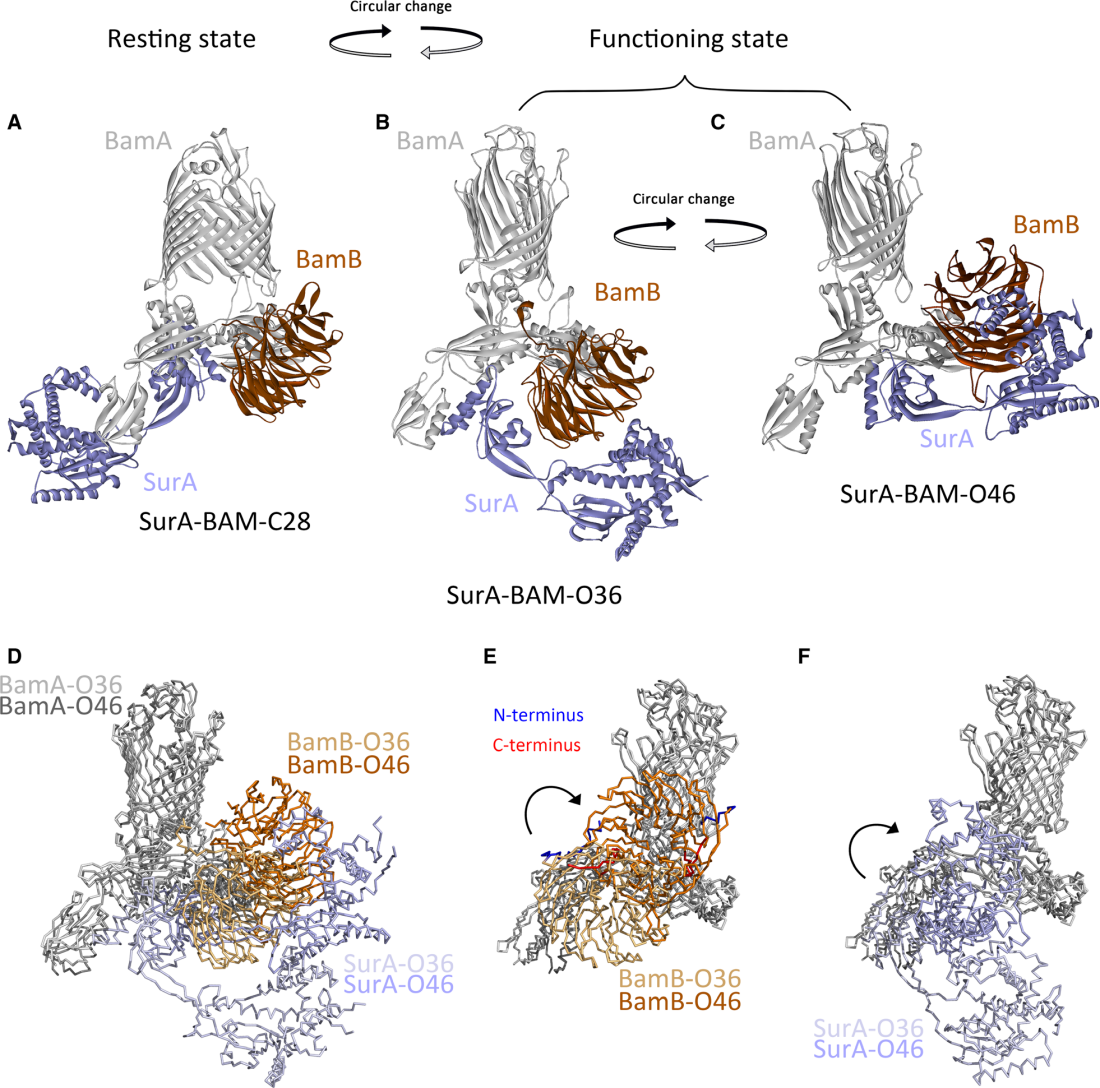
The conformational change of the SurA–BAM complex. (A–C) Shown were different conformations for the SurA–BAM complex. The SurA–BAM complex was in the ‘resting state’ in (A), and in the ‘functioning state’ in (B) and (C). Solely BamA (gray), BamB (brown) and SurA (light purple) were displayed. The black arrow indicated the circular conformational change. (D) Shown is the alignment of the indicated two models for the SurA–BAM complex (B and C) in the ‘functioning state’. Solely BamA, BamB and SurA were displayed. BamA, BamB and SurA were colored light gray, light brown and light purple in SurA–BAM–O36, but colored gray, brown and purple in SurA–BAM–O46. (E, F) Shown were the rotation and movement of the BamB subunit, and SurA as indicated by the black arrows. The N terminus (blue) and C terminus (red) of BamB in the SurA–BAM–O36 and SurA–BAM–O46 models were indicated. The structures in (D) were rotated 90 degrees and shown in (E) and (F). Solely the BamA and BamB subunits were displayed in (E), while solely BamA and SurA were displayed in (F). BamA, BamB and SurA were colored light gray, light brown and light purple in SurA–BAM–O36, but colored gray, brown and purple in SurA–BAM–O46.

The mechanism for the supercomplex in the biogenesis of OMPs was proposed according to the earlier results as illustrated in Fig. 7. The supercomplex is in the resting state without the binding of nascent OMPs (Fig. 7A). In the resting state, the SurA–BAM complex could adopt different conformations. Solely the SurA–BAM–C28 was displayed as an example. After the nascent OMPs binding, the supercomplex undergoes the conformational change to the functioning state (Fig. 7B,C). During this process, BamA opens laterally, and SurA rotates from the position in A to that in B and C (Fig. 7). In the functioning state, the supercomplex may circularly change between two conformations (Fig. 7B,C). The BamB subunit rotates and moves between the inner and the outer membrane (Fig. 7D,E). The P2 domain of SurA is docked to the BAM complex, while the N domain of SurA moves between the two membranes (Fig. 7D,F). BamB and SurA approach to the inner membrane to recognize and bind the newly translocated region of the nascent OMPs (Fig. 7B), and then move up to reach the outer membrane, facilitating the transport of the translocated part of OMPs to the outer membrane along the periplasmic domains of BamA (Fig. 7C). Moreover, BamB and SurA may also be involved in the folding and membrane integration of OMPs into the outer membrane, cooperating with the C‐terminal region of BamA. Then they move down to reach the inner membrane again and bind to the next part of the OMPs that were just translocated; thus, the entire polypeptide of the nascent OMPs is targeted to the outer membrane through this stepwise process.

The modeling data demonstrated how the conformation of the part of the supercomplex would be changed, particularly how the BamB subunit and the primary chaperone SurA behaved during the biogenesis of OMPs. The circular rotation and moving of BamB and SurA are synchronized. Meanwhile, the consequences of their behaviors are identical, which facilitates the transport of OMPs from the inner membrane to the outer membrane and maybe the folding and membrane insertion of OMPs into the outer membrane, suggesting their close cooperation in the supercomplex and their similar roles in the biogenesis of OMPs. In addition, BamB could regulate the interaction between BamA and SurA and stabilize the supercomplex in particular conformations. Thus, their functions could be partially compensated by each other, but both are required for the supercomplex to gain the entire function and high efficiency in the biogenesis of OMPs. It may interpret why deleting either BamB or SurA solely resulted in defects in the biogenesis of certain OMPs. But if both BamB and SurA are missing, it would be hard to image how the client proteins pass through the periplasm along the POTRA domains of BamA efficiently. Indeed, deleting BamB in the SurA mutant strain caused synthetic lethal phenotype [42].

The experimental and modeling results also demonstrated the interaction network between components in the supercomplex, which will make the association of the supercomplex less versatile when some of the components were lost or changed. The C‐terminal region of BamA is essential and highly conserved, mainly because of its significant function as a channel for the client proteins. The periplasmic chaperones and other BAM subunits are commonly not essential and less conserved, but they may play conserved functions like SurA and BamB. Although their substrate preferences were not identical, there is still redundancy among them. It could be one of the reasons the knockout of *surA*, *bamB*, *bamC*, *bamE* (*bamD* is essential in *E. coli*) or some of the POTRA domains was tolerable, but double knockout usually resulted in severe defects or even the lethal phenotype. Their lineage specificity makes the supercomplex recruit and accommodates different types of clients in different species. Such a mechanism will provide both the conservation and the diversity for the supercomplex, making sure that it is able to form and serve a conserved function in different species.

## Materials and methods

### Construction of bacteria strains and plasmids

Bacteria strains and plasmids used in this work are listed in Tables S1 and S2. The LY928 strain was generated for pBpa incorporation purposes, as described previously [25]. The *surA* or *bamB* knockout strain was constructed by modifying the genome of LY928 strain and designated as LY928‐∆*surA*, LY928‐∆*bamB* strain. The *E. coli* genomic DNA was used as a template to isolate the gene (including its promoter) fragment via PCR; the restriction enzyme‐free cloning [45] was used to insert the DNA fragment into the pYLC plasmid vector to construct the pYLC‐Avi‐BamA or pYLC‐BamB plasmid. The pYLC plasmid is a low‐copy plasmid derived from the pDOC plasmid [25]. The phusion site‐directed mutagenesis kit (New England Biolabs, Ipswich, MA, USA) was used to replace a certain codon by the TAG amber codon in a particular gene, through which a pBpa residue was introduced at the specific residue position. The *E. coli* strain expressing the indicated pBpa variants of BamA was constructed by replacing the *bamA* gene with the *bamA* mutant gene.

### The *in vivo* protein photo‐cross‐linking mediated by pBpa

The pYLC‐BamA or pYLC‐BamB plasmid harboring an introduced TAG codon in the *bamA* or *bamB* gene was expressed in the LY928, LY928‐∆*bamB* or LY928‐∆*surA* cells, respectively. The cells were cultured in the LB medium containing pBpa (200 μm) at 37 °C and grown to the mid‐log phase (with a *D*_600_ of ~0.8–1.0). Next, the cells were irradiated under the UV light (365 nm) for 10 min in a Hoefer UVC‐500 cross‐linker. Subsequently, the cells irradiated with the UV light were harvested by centrifugation, resuspended in SDS/PAGE loading buffer and boiled for 5 min. The protein samples were resolved by SDS/PAGE and then subjected to blotting analysis.

### Mass spectrometry analysis

The photo‐cross‐linked products of BamA‐D132pBpa and BamA‐V209pBpa variants were purified by affinity chromatography with the streptavidin resin. The eluted samples were resolved by SDS/PAGE before being subjected to the Coomassie Blue staining or the blotting analysis. The protein bands ~170 kDa were then excised from the gel and sent for liquid chromatography–tandem mass spectrometry identification as described previously [25].

### Homology models for SurA were built using the Modeler package

Experimentally determined structures for SurA have been reported, but some residues were missing in these structures. So the homology models for SurA were built using the Modeler package in a similar process described previously [26]. Protein structures related to SurA sequences were searched in the PDB_nr95 database through the BLAST Search protocol, which is based on the blastall program from Altschul et al. [46]. Protein sequences collected in the PDB_nr95 database have a 3D structure. Proper structures of SurA‐related proteins were selected as templates and loaded from the server to generate their homology models through similar processes as described by Jin [26].

### The Ramachandran plot and the Profiles‐3D were used to verify the generated homology models of SurA

The Ramachandran plot [47] was generated to check whether the residues are correctively built. It indicates conformations of the local backbone of each residue. The predicted torsion angles on either side of the alpha carbons (represented by φ and ψ) of each residue were displayed as points. The points, colored green or red to distinguish the favorable or unfavorable residues, were included in the favorable or unfavorable regions, respectively.

The Profiles‐3D could assess the compatibility of a sequence with a 3D structure [48]. It represents the 3D structure in profile scores related to the residue environments. The Verify Score is the sum of the score of each residue, which can verify the overall quality of the predicted protein structure. The results were displayed in solid ribbon style with variations in ribbon width and spectrum color regarded to the Verify Score of each residue. The structure with Verify Score higher than the Expected High Score (calculated based on the high‐resolution structures in the PDB) or between the Expected High and Low Score (45% of the Expected High Score) is mostly correct. The structure with Verify Score lower than the Expected Low Score would be grossly misfolded.

### Models for the SurA–BAM complex were constructed using the ZDOCK algorithm

The method used here was similar to that used in the previous article [26]. The ZDOCK algorithm was developed by Chen and Weng [49] to predict a protein complex through the pairwise shape complementarity method [50]. The obtained poses were clustered according to the position of ligands. The poses could be filtered through setting the residues in or outside the binding interface by users according to experimental results or rational analysis. The relationship among properties, such as the ZDOCK score, density and cluster of poses, could be explored through making 3D point plots.

To gain more successive prediction for protein complex, the poses can be reranked by the ZRANK scoring program [51], which uses the more detailed energy function [51]. ZRANK is rapid and accurate enough. Then, poses with a high density, a high ZDOCK score and a low ZRANK score were selected.

## Conflict of interest

The authors declare no conflict of interest.

## Author contributions

FJ designed and performed the experiments and the protein modeling, analyzed the data and prepared the manuscript.

## Supporting information


**Fig S1.** SurA bound to the POTRA1 domain of BamA, meanwhile, the crosslinked BamA‐SurA and BamB‐SurA were not detected in the LY928‐∆surA strain.

**Fig S2.** The binding regions for SurA and BamB were partially overlapped around the residue 135 of BamA.

**Fig S3.** Homology models for SurA were built and verified.

**Fig S4.** Filtered poses obtained by docking the indicated structures and/or models using the ZDOCK algorithm were displayed with the 3D plot.

**Fig S5.** The theoretical structures for the SurA‐BamACDE complex were predicted.

**Table S1.** *E. coli* strains used in this work.
**Table S2.** Plasmids used in this work.
**Table S3.** Mass spectrometry analysis of the cross‐linked products (around ~ 170 kD) in the BamA‐D132pBpa variant.
**Table S4.** The amount of cross‐linked products.
**Table S5.** The main‐chain RMSD and number of overlapping residues between SurA_01 and the templates.
**Table S6.** Generated models for SurA sorted by the PDF Total Energy.
**Table S7.** Verifications of SurA_01 with the Profiles‐3D.
**Table S8.** Properties of the theoretical structures for the SurA‐BAM complex in the “close” conformation.
**Table S9.** Properties of the theoretical structures for the SurA‐BAM complex in the “open” conformation.
**Table S10.** Mass spectrometry analysis of the crosslinked products (around ~ 170 kD) in the BamA‐V209pBpa variant.
**Table S11.** Properties of the theoretical structures for the BAM complex in new conformations.
**Table S12.** Properties of the theoretical structures for the SurA‐BAM complex in the new conformations.

## Data Availability

The protein structures used for modeling in this work are openly available in PDB (http://www.rcsb.org). The accession number for the structure of the BAM complex in the ‘close’ conformation is: 5AYW. The accession number for the structure of the BAM complex in the open conformation is: 5LJO. The accession number for the structure of the BamACDE complex in the close conformation is: 5EKQ. The accession numbers for the structure of SurA are: 1M5Y and 3PV1. The protein sequences of BamA, BamB and SurA in *E. coli* are openly available in UniProt (https://www.uniprot.org) with the accession numbers P0A940, P77774 and P0ABZ6. The protein sequences used for analyzing the conservation of residues in BamA, BamB and SurA in this work are openly available in the Uniref90 database, which is downloaded from UniProt (https://www.uniprot.org/downloads) and installed in the Discovery Studio Software. The raw data are available from the corresponding author upon reasonable request.
