# Supplementary figures and images for: The transmembrane supercomplex mediating the biogenesis of OMPs in Gram‐negative bacteria assumes a circular conformational change upon activation
Source: FEBS Open Bio. 2020 Jul 23;10(8):1698–715. doi: 10.1002/2211-5463.12922 (PMC7396438; doi:10.1002/2211-5463.12922)

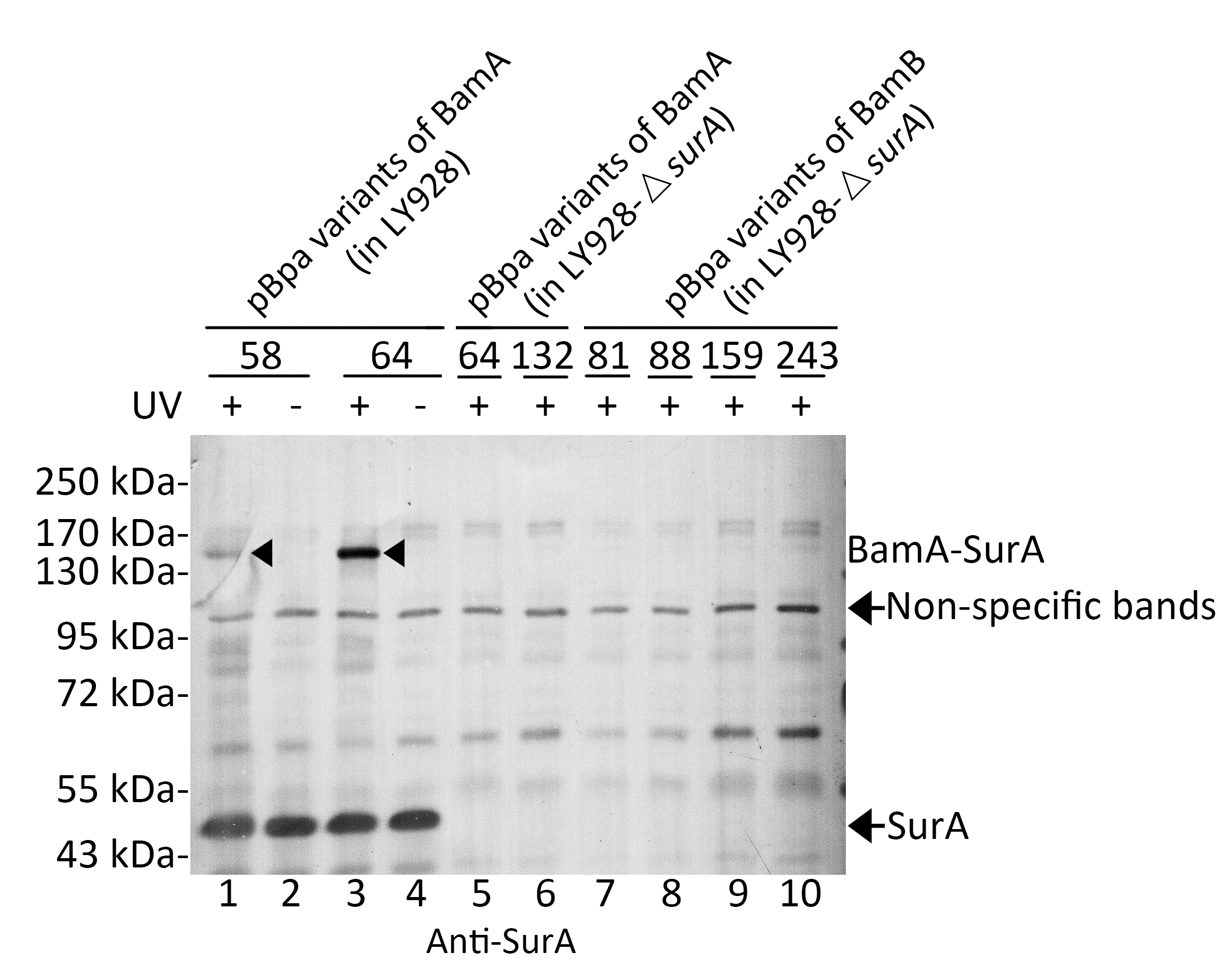

Supplement: Supplementary file 1 — Fig S1. SurA bound to the POTRA1 domain of BamA, meanwhile, the crosslinked BamA‐SurA and BamB‐SurA were not detected in the LY928‐∆surA strain. [file FEB4-10-1698-s001.tif]

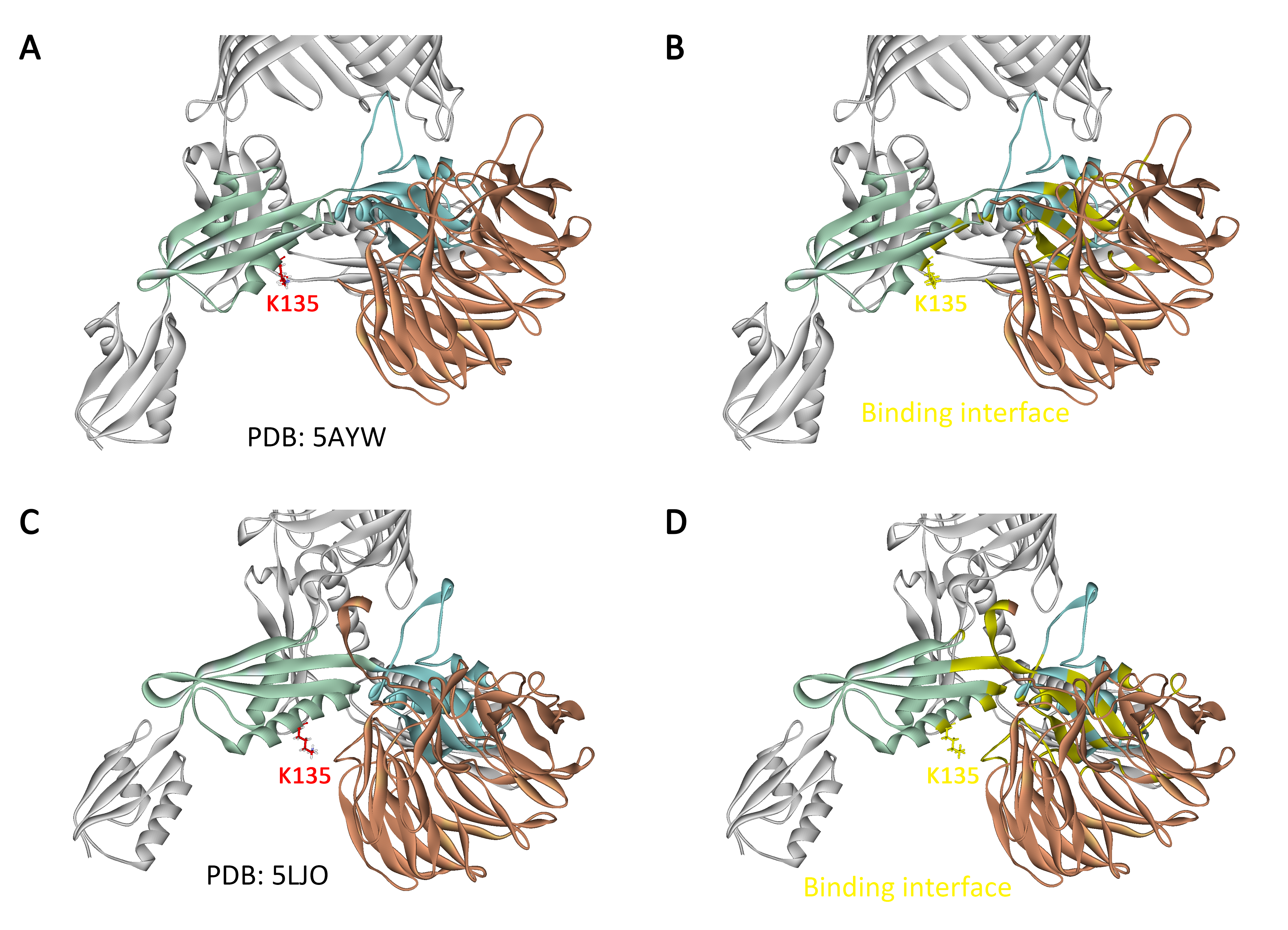

Supplement: Supplementary file 2 — Fig S2. The binding regions for SurA and BamB were partially overlapped around the residue 135 of BamA. [file FEB4-10-1698-s002.tif]

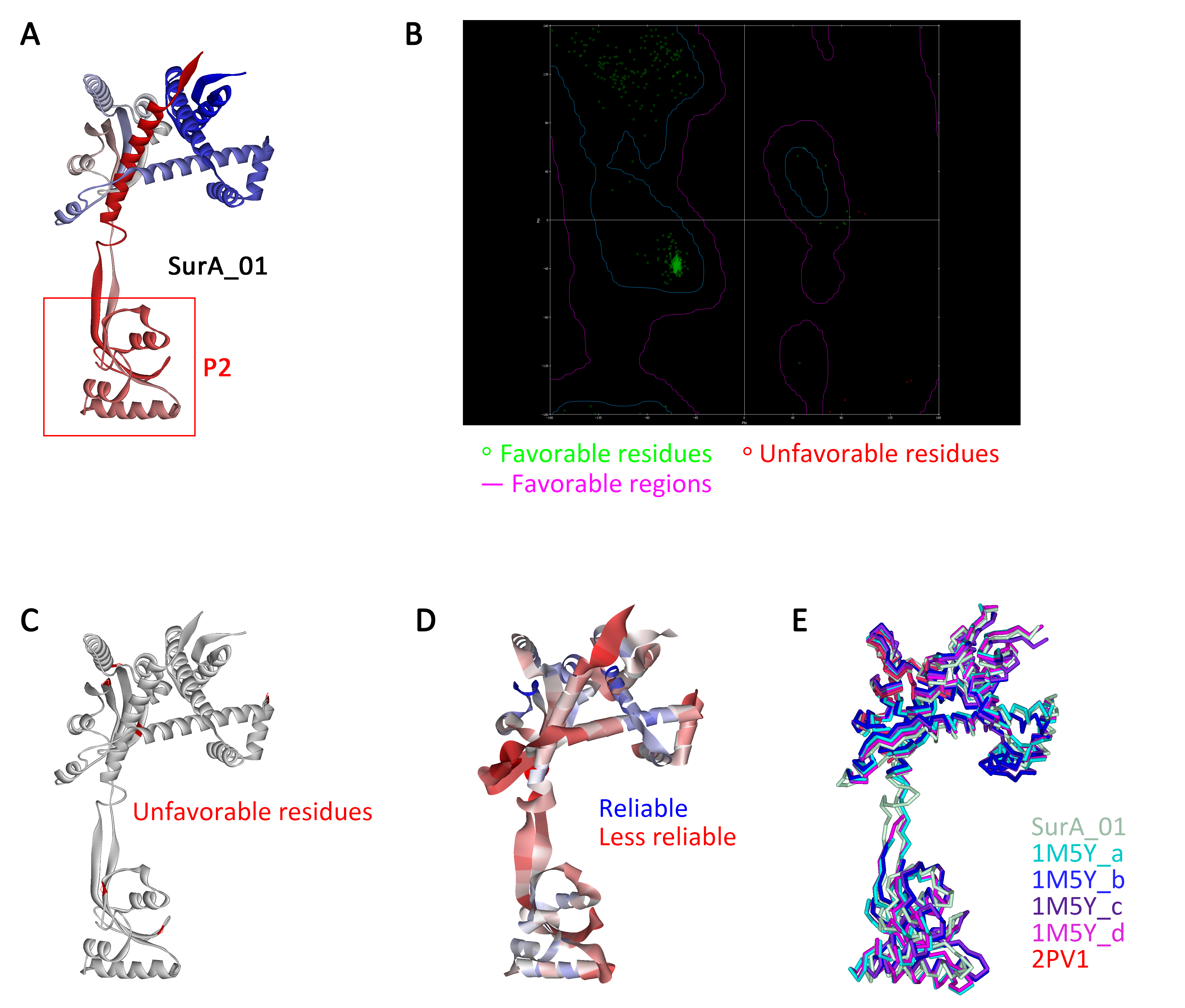

Supplement: Supplementary file 3 — Fig S3. Homology models for SurA were built and verified. [file FEB4-10-1698-s003.tif]

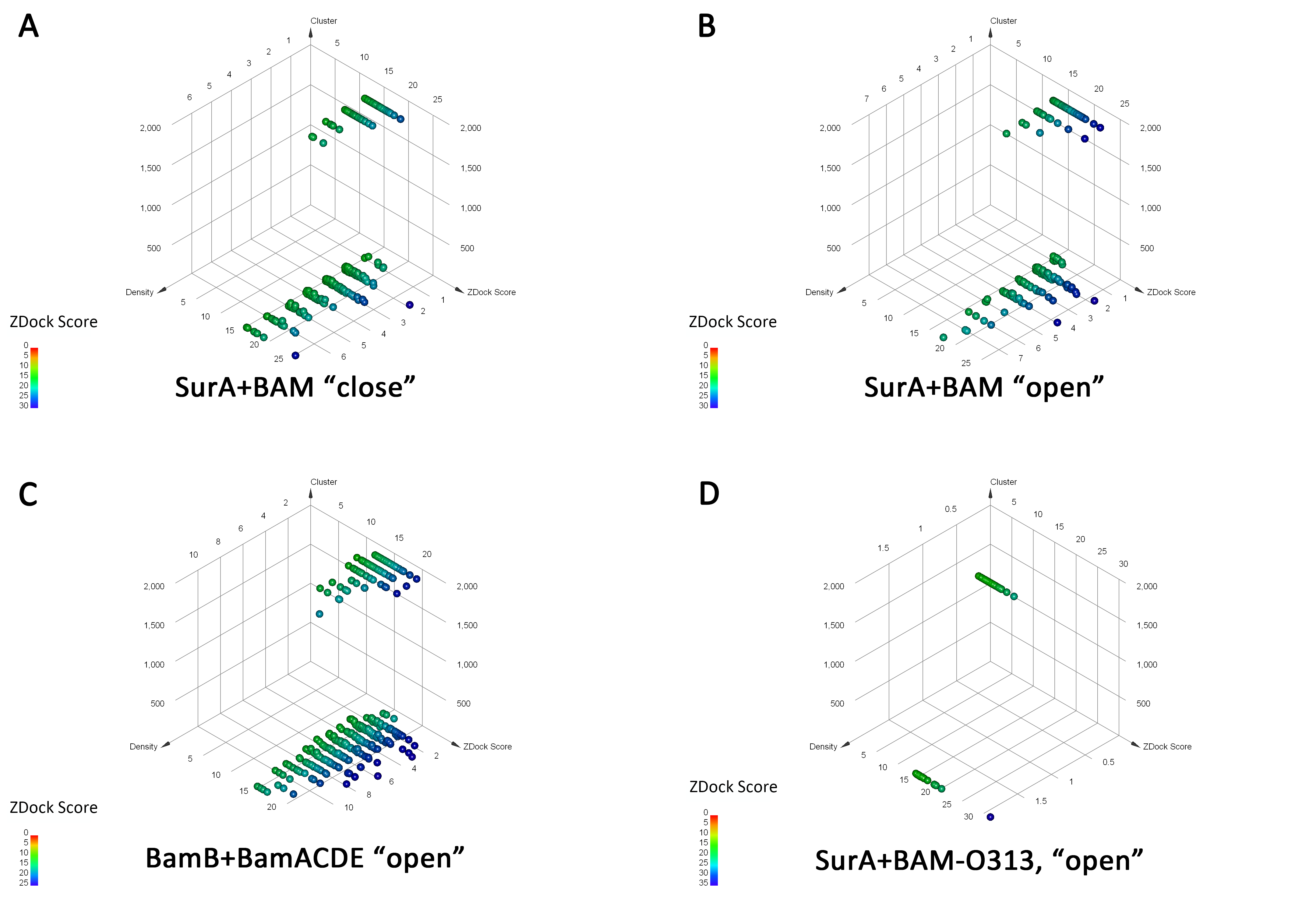

Supplement: Supplementary file 4 — Fig S4. Filtered poses obtained by docking the indicated structures and/or models using the ZDOCK algorithm were displayed with the 3D plot. [file FEB4-10-1698-s004.tif]

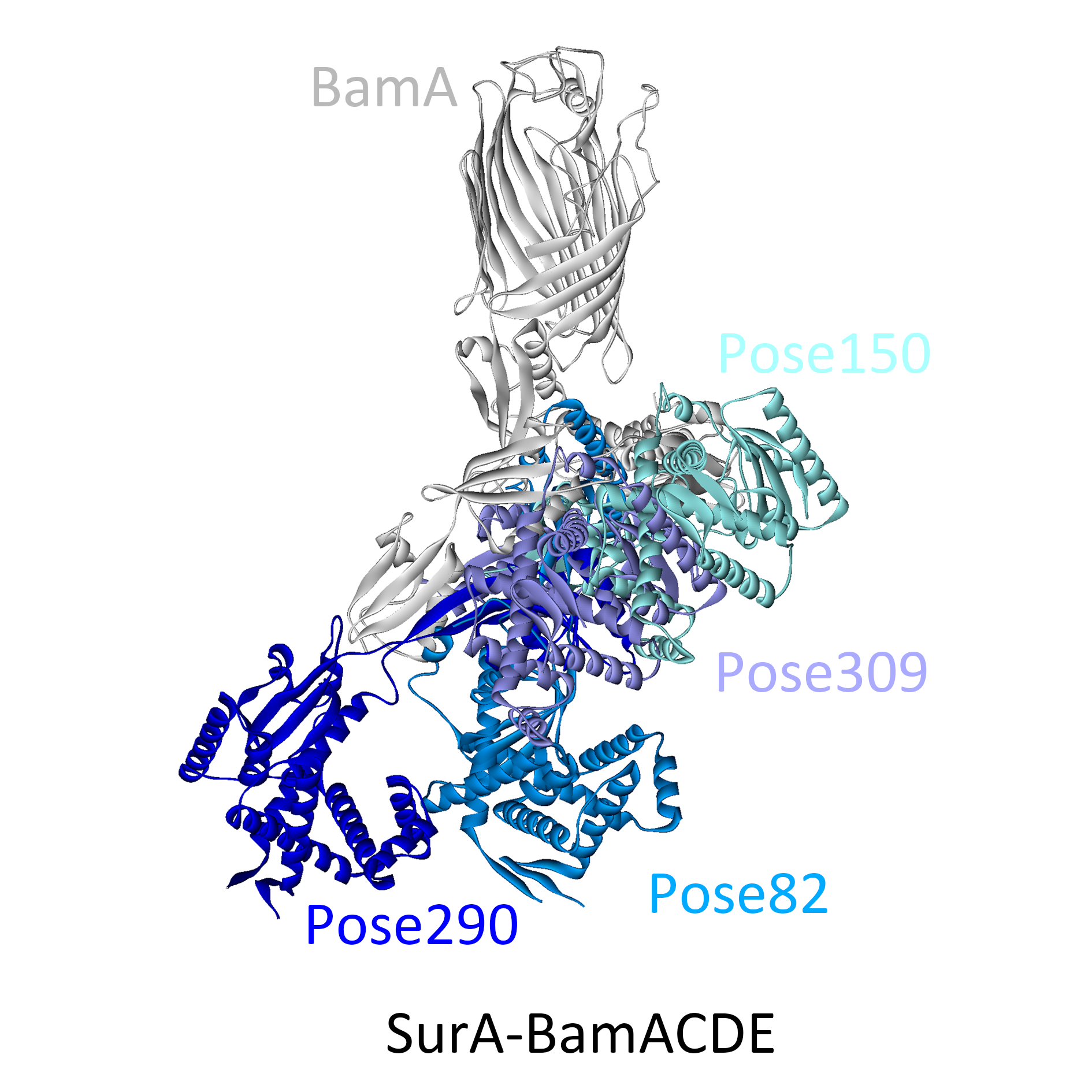

Supplement: Supplementary file 5 — Fig S5. The theoretical structures for the SurA‐BamACDE complex were predicted. [file FEB4-10-1698-s005.tif]
