# Supplementary material for: The transmembrane supercomplex mediating the biogenesis of OMPs in Gram‐negative bacteria assumes a circular conformational change upon activation
Source: FEBS Open Bio. 2020 Jul 23;10(8):1698–715. doi: 10.1002/2211-5463.12922 (PMC7396438; doi:10.1002/2211-5463.12922)
Supplement: Supplementary file 6 — Table S1. E. coli strains used in this work. Table S2. Plasmids used in this work. Table S3. Mass spectrometry analysis of the cross‐linked products (around ~ 170 kD) in the BamA‐D132pBpa variant. Table S4. The amount of cross‐linked products. Table S5. The main‐chain RMSD and number of overlapping residues between SurA_01 and the templates. Table S6. Generated models for SurA sorted by the PDF Total Energy. Table S7. Verifications of SurA_01 with the Profiles‐3D. Table S8. Properties of the theoretical structures for the SurA‐BAM complex in the “close” conformation. Table S9. Properties of the theoretical structures for the SurA‐BAM complex in the “open” conformation. Table S10. Mass spectrometry analysis of the crosslinked products (around ~ 170 kD) in the BamA‐V209pBpa variant. Table S11. Properties of the theoretical structures for the BAM complex in new conformations. Table S12. Properties of the theoretical structures for the SurA‐BAM complex in the new conformations. [file FEB4-10-1698-s006.docx]

**Supplemental tables**

**Table S1. *E. coli* strains used in this work**

| **Strain** | **Relevant genotype (and reference)** | **Source** |
| --- | --- | --- |
| **BW25113** | Wild type *E. coli* cells [[1](#_ENREF_1)] | Keio collection, Japan |
| **LY928** | The modified BW25113 strain, in which the genome was modified to encode the orthogonal amino-acyl tRNA synthetase and the tRNA required for pBpa incorporation [[2](#_ENREF_2)] | Yang Liu and Jiayu Yu |
| **LY928-Avi-BamA** | The modified LY928 strain, in which the *bamA* gene was modified to encode the wild type BamA protein containing an Avi-tag at the N-terminus. | This work |
| **LY928-Avi-BamA-R64pBpa**  **LY928-Avi-BamA-D132pBpa**  **LY928-Avi-BamA-R64&D132pBpa**  **LY928-Avi-BamA-K135pBpa**  **LY928-Avi-BamA-V209pBpa** | The modified LY928 strain, in which the *bamA* gene was replaced by the *bamA* mutants. | This work |
| **LY928-∆*bamB*** | Modified LY928 strain, in which the *bamB* gene was deleted | This work |
| **LY928-∆*surA*** | Modified LY928 strain, in which the s*urA* gene was deleted | This work |

**Table S2. Plasmids used in this work.**

| **Plasmid** | **Description** | **Source** |
| --- | --- | --- |
| **pYLC** | A low copy plasmid derived from the pDOC plasmid with a mutation in the replication origin [[2](#_ENREF_2)] and expressing the ampicillin resistance gene | Yang Liu and Jiayu Yu |
| **pYLC-Avi-BamA** | For expressing the wild type BamA protein that contains an Avi-tag at the N-terminus (controlled by its natural promoter). | This work |
| **pYLC-Avi-BamA-D58pBpa**  **pYLC-Avi-BamA-R64pBpa**  **pYLC-Avi-BamA-V121pBpa**  **pYLC-Avi-BamA-E123pBpa**  **pYLC-Avi-BamA-T129pBpa**  **pYLC-Avi-BamA-D132pBpa**  **pYLC-Avi-BamA-K135pBpa**  **pYLC-Avi-BamA-D139pBpa**  **pYLC-Avi-BamA-V209pBpa** | For expressing the indicated pBpa variants of the BamA protein that contains an Avi-tag at the N-terminus (controlled by its natural promoter) | This work |
| **pYLC-BamB** | For expressing the wild type BamB protein (controlled by its natural promoter) | This work |
| **pYLC-BamB-V81pBpa**  **pYLC-BamB-N85pBpa**  **pYLC-BamB-D88pBpa**  **pYLC-BamB-V131pBpa**  **pYLC-BamB-D159pBpa**  **pYLC-BamB-E176pBpa**  **pYLC-BamB-R243pBpa**  **pYLC-BamB-S245pBpa** | For expressing the pBpa variants of BamB with the indicated residue replacement (controlled by its natural promoter) | This work |

**Table S3. Mass spectrometry analysis of the cross-linked products (around ~ 170 kD) in the BamA-D132pBpa variant.**

| **Protein** | **Score in the crosslinked sample** | **Score in the negative control** |
| --- | --- | --- |
| **BamA** | **633** | **99** |
| **SurA** | **413** | **-** |

**Table S4. The amount of cross-linked products**

|  |  | **IntDen** | | | | |
| --- | --- | --- | --- | --- | --- | --- |
|  | Repeats | Daul-64 | Daul-132 | Daul-64&132 | 64 | 132 |
| **BamA-SurA** | 1 | 166405 | 164860 | 331265 | 369924 | 317890 |
|  | 2 | 152970 | 157266 | 310236 | 462037 | 393403 |
| **Control**  **(The non-specific band)** | 1 | 184141 | 184141 | 184141 | 215203 | 164482 |
|  | 2 | 138285 | 138285 | 138285 | 207955 | 169842 |
| **BamA-SurA/Control** | 1 | 0.90 | 0.90 | 1.80 | 1.72 | 1.93 |
|  | 2 | 1.11 | 1.14 | 2.24 | 2.22 | 2.32 |
| **Average** |  | 1.00 | 1.02 | 2.02 | 1.97 | 2.12 |
| **Standard deviation** |  | 0.14 | 0.17 | 0.31 | 0.36 | 0.27 |

**Table S5. The main-chain RMSD and number of overlapping residues between SurA_01 and the templates**

|  | **SurA_ 01** | **1M5Y_a** | **1M5Y_b** | **1M5Y_c** | **1M5Y _d** | **2PV1** |
| --- | --- | --- | --- | --- | --- | --- |
| **SurA_01** |  | 384 | 374 | 373 | 385 | 103 |
| **1M5Y _a** | 2.834 Å |  | 372 | 371 | 387 | 103 |
| **1M5Y _b** | 3.75 Å | 2.535 Å |  | 376 | 373 | 103 |
| **1M5Y _c** | 3.223 Å | 2.461 Å | 0.844 Å |  | 372 | 102 |
| **1M5Y _d** | 2.279 Å | 1.098 Å | 2.896 Å | 2.592 Å |  | 103 |
| **2PV1** | 0.647 Å | 1.307 Å | 1.884 Å | 1.479 Å | 0.973 Å |  |

**Table S6. Generated models for SurA sorted by the PDF Total Energy**

| **Model Name** | **PDF Total Energy** | **PDF Physical Energy** | **DOPE Score** |
| --- | --- | --- | --- |
| **SurA_ 01** | 5274.68 | 997.85 | -39312.62 |
| **SurA_05** | 5386.99 | 1014.58 | -38967.89 |
| **SurA_02** | 5421.56 | 1012.88 | -38799.88 |
| **SurA_03** | 5568.7 | 1155.57 | -38536.1 |
| **SurA_04** | 6364.25 | 1149.26 | -38896.79 |

**Table S7. Verifications of SurA_01 with the Profiles-3D**

| **Verify Score** | **Verify Expected High Score** | **Verify Expected Low Score** |
| --- | --- | --- |
| 167.31 | 184.27 | 82.92 |

**Table S8. Properties of the theoretical structures for the SurA-BAM complex in the “close” conformation**

| **Pose** | **Cluster** | **Density** | **ZDOCK Score** | **ZRANK Score** | **Binding site residues** | | **Conformation** |
| --- | --- | --- | --- | --- | --- | --- | --- |
|  |  |  |  |  | 159 | 243 |  |
| **1** | 9 | 5 | 19.1 | -86.605 | + | + | 2 |
| **2** | 47 | 2 | 19.34 | -79.375 |  | + | 2 |
| **3** | 24 | 3 | 18.16 | -75.179 |  | + | 2 |
| **11** | 26 | 3 | 23.14 | -52.026 |  | + | 2 |
| **19** | 3 | 4 | 17.74 | -39.501 |  | + | 2 |
| **28** | 55 | 3 | 15.94 | -31.318 |  | + | 1 |
| **49** | 9 | 4 | 17.78 | -10.132 | + |  | 2 |
| **53** | 24 | 2 | 17.68 | -9.35 | + | + | 2 |
| **56** | 44 | 2 | 20.88 | -7.574 |  | + | 3 |
| **57** | 14 | 2 | 19.66 | -4.621 |  | + | 2 |
| **60** | - | 3 | 15.26 | -2.558 | + | + | 2 |

**Table S9. Properties of the theoretical structures for the SurA-BAM complex in the “open” conformation**

| **Pose** | **Cluster** | **Density** | **ZDOCK Score** | **ZRANK Score** | **Residues** | | **Conformation** |
| --- | --- | --- | --- | --- | --- | --- | --- |
|  |  |  |  |  | 159 | 243 |  |
| **30** | 7 | 4 | 15.84 | -39.474 |  |  | 4 |
| **90** | 7 | 4 | 15.96 | -11.399 |  |  | 4 |
| **73** | 17 | 3 | 19.4 | -17.663 |  |  | 5 |
| **57** | 17 | 3 | 17.72 | -23.273 |  |  | 5 |
| **7** | 28 | 2 | 16.12 | -61.196 |  |  | 1 |
| **38** | - | 2 | 16.02 | -36.384 |  |  | 2 |
| **36** | 30 | 2 | 16.44 | -36.445 | + |  | 2 |
| **95** | 28 | 2 | 16.4 | -9.062 |  |  | 1 |

**Table S10. Mass spectrometry analysis of the crosslinked products (around ~ 170 kD) in the BamA-V209pBpa variant.**

| **Protein** | **Score in the crosslinked sample 1** | **Score in the crosslinked sample 2 (repeated)** | **Score in the negative control** |
| --- | --- | --- | --- |
| **BamA** | **2076** | **2031** | **-** |
| **BamB** | **924** | **752** | **-** |

**Table S11. Properties of the theoretical structures for the BAM complex in new conformations**

| **Pose** | **Cluster** | **Density** | **ZDOCK Score** |
| --- | --- | --- | --- |
|  |  |  |  |
| **200** | - | 2 | 16.38 |
| **313** | 2 | 2 | 14.76 |

**Table S12. Properties of the theoretical structures for the SurA-BAM complex in the new conformations**

| **Pose** | **Cluster** | **Density** | **ZDOCK Score** | **Residues in BamA** | | | **Residues in BamB** | |
| --- | --- | --- | --- | --- | --- | --- | --- | --- |
|  |  |  |  | 64 | 132 | 135 | 159 | 243 |
| **5** | 3 | 2 | 18.86 | 18Å | 7.6 Å | + | + |  |
| **7** | 4 | 2 | 18.46 | 18Å | 6.3 Å | + | + |  |
| **37** | 3 | 2 | 14.98 | 17Å | 9.2 Å | + | + |  |
| **46** | 4 | 2 | 14.76 | 13Å | + | + | + |  |

**References**

1. Baba, T. & Mori, H. (2008) The construction of systematic in-frame, single-gene knockout mutant collection in Escherichia coli K-12, *Methods in molecular biology.* **416**, 171-81.

2. Wang, Y., Wang, R., Jin, F., Liu, Y., Yu, J., Fu, X. & Chang, Z. (2016) A Supercomplex Spanning the Inner and Outer Membranes Mediates the Biogenesis of beta-Barrel Outer Membrane Proteins in Bacteria, *The Journal of biological chemistry.* **291**, 16720-9.
